# Supplementary material for: Mechanistic insights into E. coli recovery from growth arrest
Source: Nat Commun. 2026 Apr 11;17:5124. doi: 10.1038/s41467-026-71781-6 (PMC13246957; doi:10.1038/s41467-026-71781-6)
Supplement: Supplementary file 1 — Supplementary Information [file 41467_2026_71781_MOESM1_ESM.pdf]

Supplementary Information

Mechanistic insights into *E. coli* recovery from growth arrest

Ahmed H. Hassan[1*], Yuko Nakano[2*], Howard Gamper[2], Isao Masuda[2], Ludmila Vesela[1], Matyas Pinkas[1], Sathya Narayanan Nagarajan[3], Jonathan Dworkin[3], Gregor Blaha[4], Ya-Ming Hou[2#], Gabriel Demo[1#]

[1]Central European Institute of Technology, Masaryk University, Brno, Czech Republic

[2]Department of Biochemistry and Molecular Biology, Thomas Jefferson University, Philadelphia, USA

[3]Department of Microbiology and Immunology, Columbia University, New York, USA

[4]Department of Biochemistry, University of California, Riverside, California, USA

Supplementary Table 1. Primers used for strain constructions

Gene	Primer	Sequence (5′ -> 3′)	Purpose
rsfS	rsfS-F	ACCCAGGGGGAAAACTTGCAGGGTAAAGCACGTGTAGGCTGGAGCTGCTTC	Δ*rsfS* strain construction
	rsfS-R	TTCACGCATTAACTCCAGAGTTTTTCCAGTATGGGAATTAGCCATGGTCC	
	rsfS-F	GGCGACAATTGTCCCGAAATCG	*rsfS* deletion check
	rsfS-R	GGAATTTCAATCAGCTCGAAGG	
rsmE	rsmE-F	CGACGCGGATTTTTAACTATGCGTATCCCCCGCATTTATCGTGTAGGCTGGAGCTGCTTC	Δ*rsmE* strain construction
	rsmE-R	CTTCTCCGTTAGCCCAAATCGCCAAATCGTACTTGTAGCGATGGGAATTAGCCATGGTCC	
	rsmE-F	GCAGCTGTTCAACGCATGGAAC	*rsmE* deletion check
	rsmE-R	CACCATTGATCAGATACAGATCG	

Supplementary Table 2. Primers used for plasmid constructions

Mutated nucleotides are underlined.

Gene	Primer	Sequence (5′ -> 3′)	Purpose
rimM	rimM-F	GCGTGGAAAGATCTGATGGG	Y106A/Y107A in His-*rimM*
	rimM-R	AGCATCACCTTCTTCCAGCTGTG	
rbfA	rbfA-F	AAAGGGCCATGGCGAAAGAATTTGGTCG	*rbfA* cloning
	rbfA-R	AAAGGGGGATCCTTAGTCCTCCTTGCTGTCGTCC	

Supplementary Table 3

Refinement statistics for cryo-EM structures 30S$_{\Delta rimM}$ - state I, 30S$_{\Delta rimM}$ state - II, 30S$_{\Delta rimM}$ state - III, pre-50S$_{\Delta rimM}$ +RsfS (-H68), 50S$_{\Delta rimM}$ +RsfS +H68 and 70S$_{\Delta rimM}$

	**30S$_{\Delta rimM}$ class I**	**30S$_{\Delta rimM}$ class II**	**30S$_{\Delta rimM}$ class III**	**pre-50S$_{\Delta rimM}$ +RsfS -H68**	**50S$_{\Delta rimM}$ +RsfS +H68**	**70S$_{\Delta rimM}$**
**PDBID**	9SS0	9SS1	9SS2	9SS4	9SS5	9SS6
**EMBD**	EMD-55171	EMD-55173	EMD-55174	EMD-55176	EMD-55177	EMD-55178
**Data collection and processing**						
Magnification	105,000x	105,000x	105,000x	105,000x	105,000x	105,000x
Voltage (kV)	300	300	300	300	300	300
Electron exposure ($e^-/Å^2$)	40	40	40	40	40	40
Defocus range (μm)	-0.5 to -2.0	-0.5 to -2.0	-0.5 to -2.0	-0.5 to -2.0	-0.5 to -2.0	-0.5 to -2.0
Pixel size (Å)	0.834	0.834	0.834	0.834	0.834	0.834
Symmetry imposed	C1	C1	C1	C1	C1	C1
Initial particle (no.)	455076	455076	455076	652894	652894	900281
Final particle (no.)	12314	39565	30144	52567	51537	101932
Map resolution (Å)	3.0	2.9	2.9	2.7	2.7	2.6
FSC threshold	0.143	0.143	0.143	0.143	0.143	0.143
**Refinement**						
Initial model used (PDB code)	6WDE	6WDE	6WDE	6WDE	6WDE	6WDE
Model resolution (Å)	3.0	3.0	3.0	3.0	3.0	3.0
Correlation Coefficient (cc_mask)*	0.84	0.89	0.88	0.91	0.92	0.90
Map sharpening B factor ($Å^2$)	-30	-30	-30	-40	-40	-50
Model composition*						
Non-hydrogen atoms	47157	49977	53770	91562	92721	144897
Protein residues	1968	2190	2630	3698	3584	6015
RNA residues	1475	1525	1539	2928	3023	4562
B factors ($Å^2$)*						
Protein	157.09	182.47	182.00	150.16	127.89	138.97
RNA	170.19	183.44	146.71	135.18	126.31	124.61
R.m.s. deviations*,§						
Bond lengths (Å)	0.004	0.004	0.004	0.004	0.004	0.004
Bond angles (°)	0.769	0.716	0.758	0.702	0.700	0.717
Validation#						
MolProbity score	1.94	1.83	1.86	1.47	1.49	1.61
Clashscore	6.26	5.01	5.45	3.06	3.10	3.36
Poor rotamers (%)	0.37	0.16	0.14	0.40	0.14	0.30
Ramachandran plot#						
Favored (%)	88.21	89.35	89.44	94.57	94.18	92.21
Allowed (%)	11.63	10.51	10.44	5.37	5.77	7.69
Disallowed (%)	0.16	0.14	0.12	0.06	0.06	0.10
Validation (RNA)#						
Good sugar pucker (%)	98.85	99.21	98.83	99.21	99.14	99.17
Good backbone (%)	80.14	82.75	84.86	84.15	83.96	85.14

* from Phenix
# from Molprobity
§ root mean square deviations

Supplementary Table 4

Refinement statistics for cryo-EM maps 30S$_{wt}$, pre-50S$_{wt}$ +RsfS (-H68), 50S$_{wt}$ +RsfS +H68 and 70S$_{wt}$

	**30S$_{wt}$**	**pre-50S$_{wt}$ +RsfS (-H68)**	**50S$_{wt}$ +RsfS +H68**	**70S$_{wt}$**
**EMBD**	EMD-55181	EMD-55182	EMD-55183	EMD-55185
**Data collection and processing**				
Magnification	105,000x	105,000x	105,000x	105,000x
Voltage (kV)	300	300	300	300
Electron exposure (e^{-}/Å^{2})	40	40	40	40
Defocus range (μm)	-0.5 to -2.0	-0.5 to -2.0	-0.5 to -2.0	-0.5 to -2.0
Pixel size (Å)	0.834	0.834	0.834	0.834
Symmetry imposed	C1	C1	C1	C1
Initial particle (no.)	826581	970120	970120	978750
Final particle (no.)	43157	60038	11115	146167
Map resolution (Å)	3.1	2.7	2.9	3.0
FSC threshold	0.143	0.143	0.143	0.143

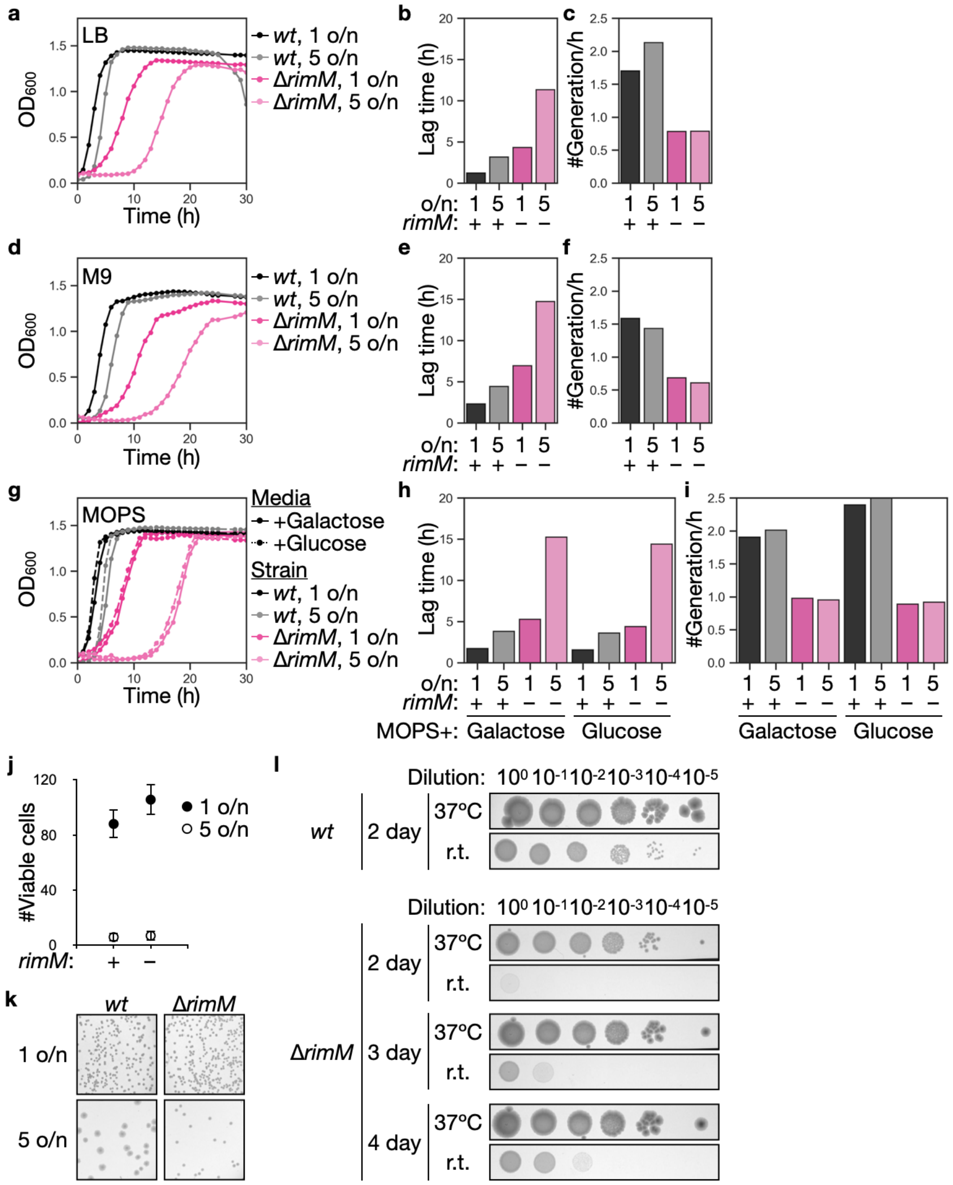

Supplementary Figure 1. Assembly of *E. coli* 30S subunit in growth recovery. (a) Growth profiles of the *wt* and Δ*rimM* strains in LB at 37ºC after 1-day or 5-day pre-culture (n = 1). **(b)** Lag time calculated from OD_{600}'s of cultures in (a), as the time required to reach OD_{600} of 0.2. **(c)** Growth rates (the number of generations per hour) of cultures in (a). **(d)** Growth profiles of *wt* and Δ*rimM* strains in M9 minimal medium at 37ºC after 1-day or 5-day pre-culture (n = 1). **(e)** Lag time calculated from OD_{600}'s of cultures in (d). **(f)** Growth rates (the number of generations per hour) of cultures in (d). **(g)** Growth profiles of *wt* and Δ*rimM* strains in MOPS rich medium supplemented with 0.2% galactose or 0.2% glucose at 37ºC after 1-day or 5-day pre-culture (n = 1). **(h)** Lag time calculated from OD_{600}'s of cultures in (g). **(i)** Growth rates (the number of generations per hour) of cultures in (g). **(j)** Viable cells of *wt* and Δ*rimM* strains (n = 6; technical replicates). Cell counts were calculated from the colony number within an area of the same size on LB plates spread after continuous liquid culture for 1 day or 5 days at 37ºC. **(k)** Zoomed-in views of *wt* and Δ*rimM* strains on LB plates after 1-day or 5-day liquid culture. **(l)** Cold sensitivity of *wt* and Δ*rimM* strains after one overnight growth in LB and incubated on LB plates at room temperature (r.t.) or 37ºC.

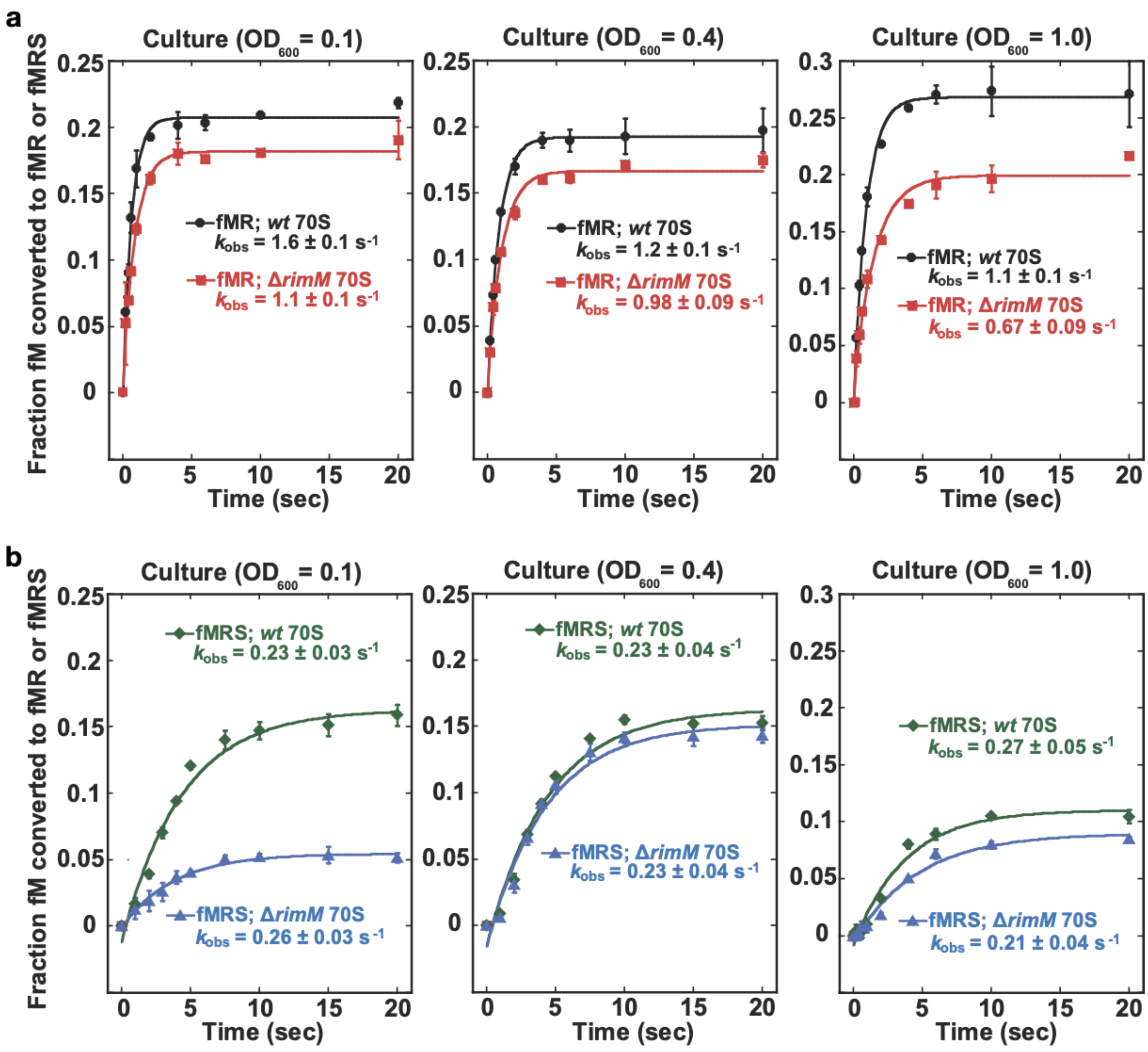

Supplementary Figure 2. Kinetics of synthesis of dipeptide and tripeptide by *E. coli wt* and Δ*rimM* ribosomes. (a) A time course of the fractional conversion of fM to fMR by Δ*rimM* and *wt* MRE600 ribosomes harvested at the indicated OD_{600}'s. Error bars, mean ± SD (n = 3). **(b)** A time course of the conversion of fM to fMRS by Δ*rimM* and *wt* ribosomes harvested at the indicated OD_{600}'s. Error bars, mean ± SD (n = 3).

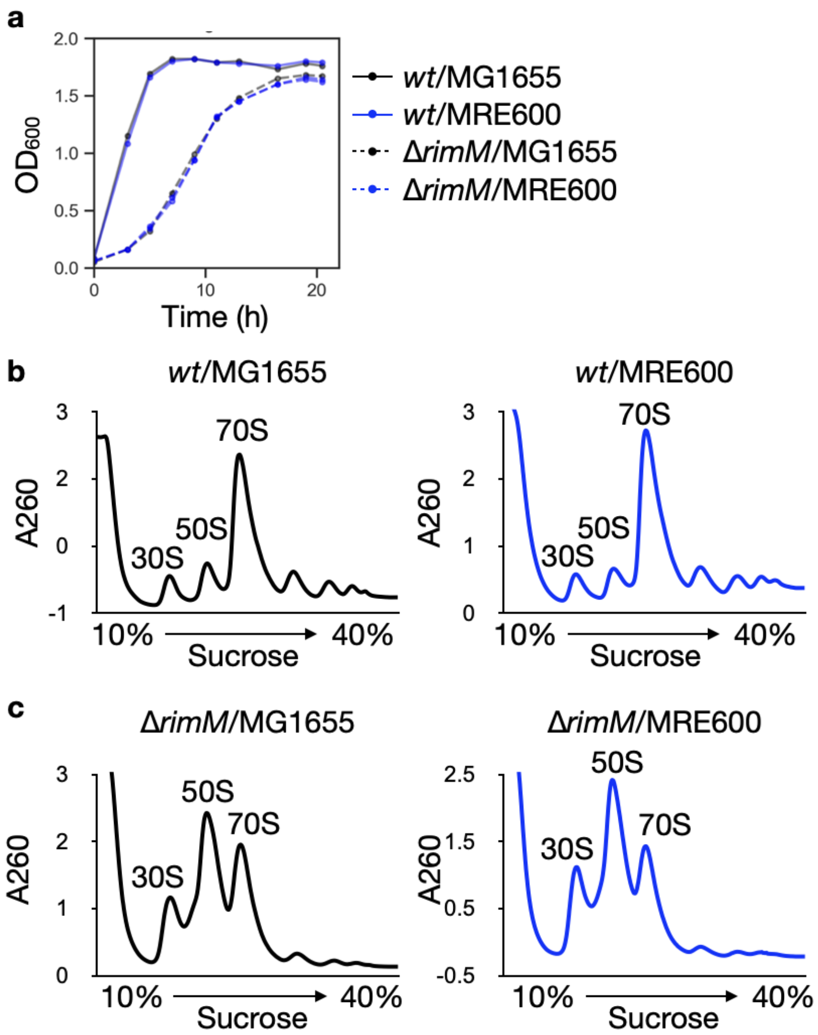

Supplementary Figure 3. Similar profiles of *E. coli* MG1655 and MRE600 strains. (a) Growth profiles of *wt* and Δ*rimM* strains in MG1655 and in MRE600 in LB (n = 1). **(b)** Polysome profiles of *wt* strains in MG1655 and in MRE600 harvested at OD_{600} 0.4 in LB (n = 1) and fractionated in a 10-40% sucrose gradient. **(c)** Polysome profiles of Δ*rimM* strains in MG1655 and in MRE600 harvested at OD_{600} 0.4 in LB (n = 1) and fractionated in a 10-40% sucrose gradient.

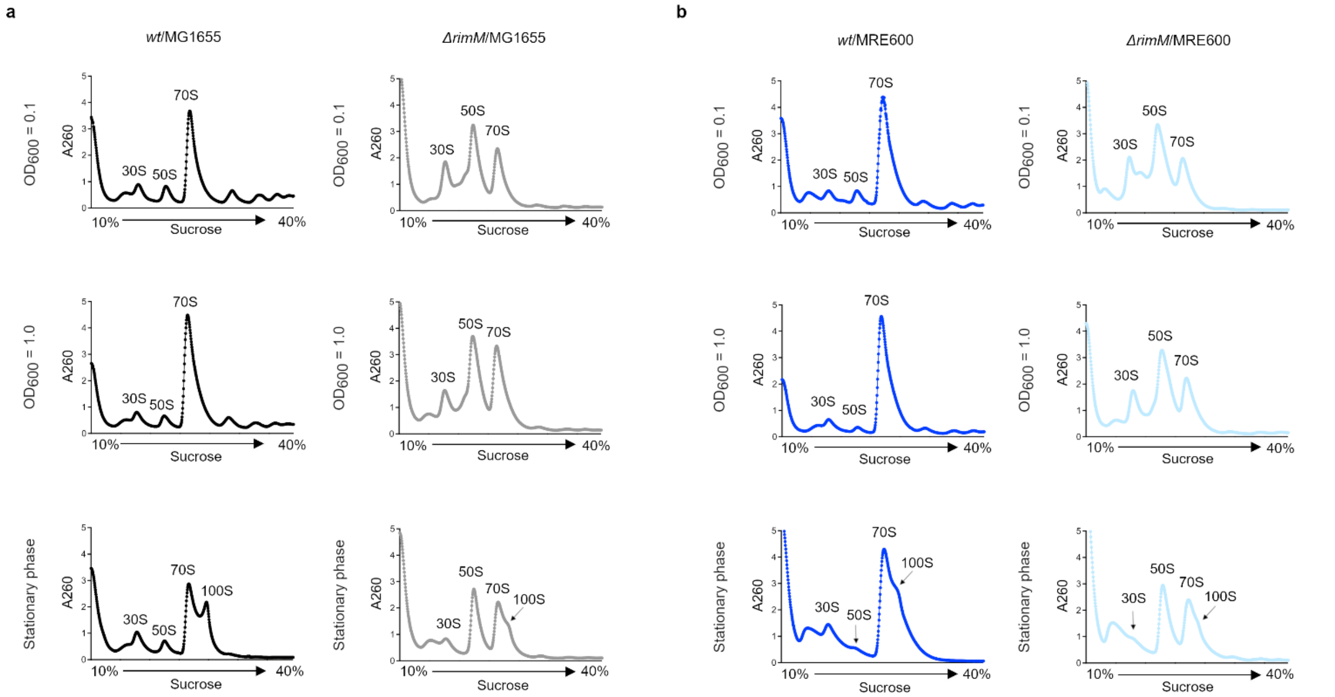

Supplementary Figure 4. Ribosome profiles of *E. coli* MG1655 and MRE600 strains in different OD_{600}. (**a**) Polysome profiles of *wt* (black) and Δ*rimM* (grey) strains in MG1655 harvested at OD_{600} 0.1, 1.0 and stationary phase in LB (n=1) and fractionated in a 10-40% sucrose gradient. (**b**) Polysome profiles of *wt* (blue) and Δ*rimM* (light blue) strains in MRE600 harvested at OD_{600} 0.1, 1.0 and stationary phase in LB (n=1) and fractionated in a 10-40% sucrose gradient.

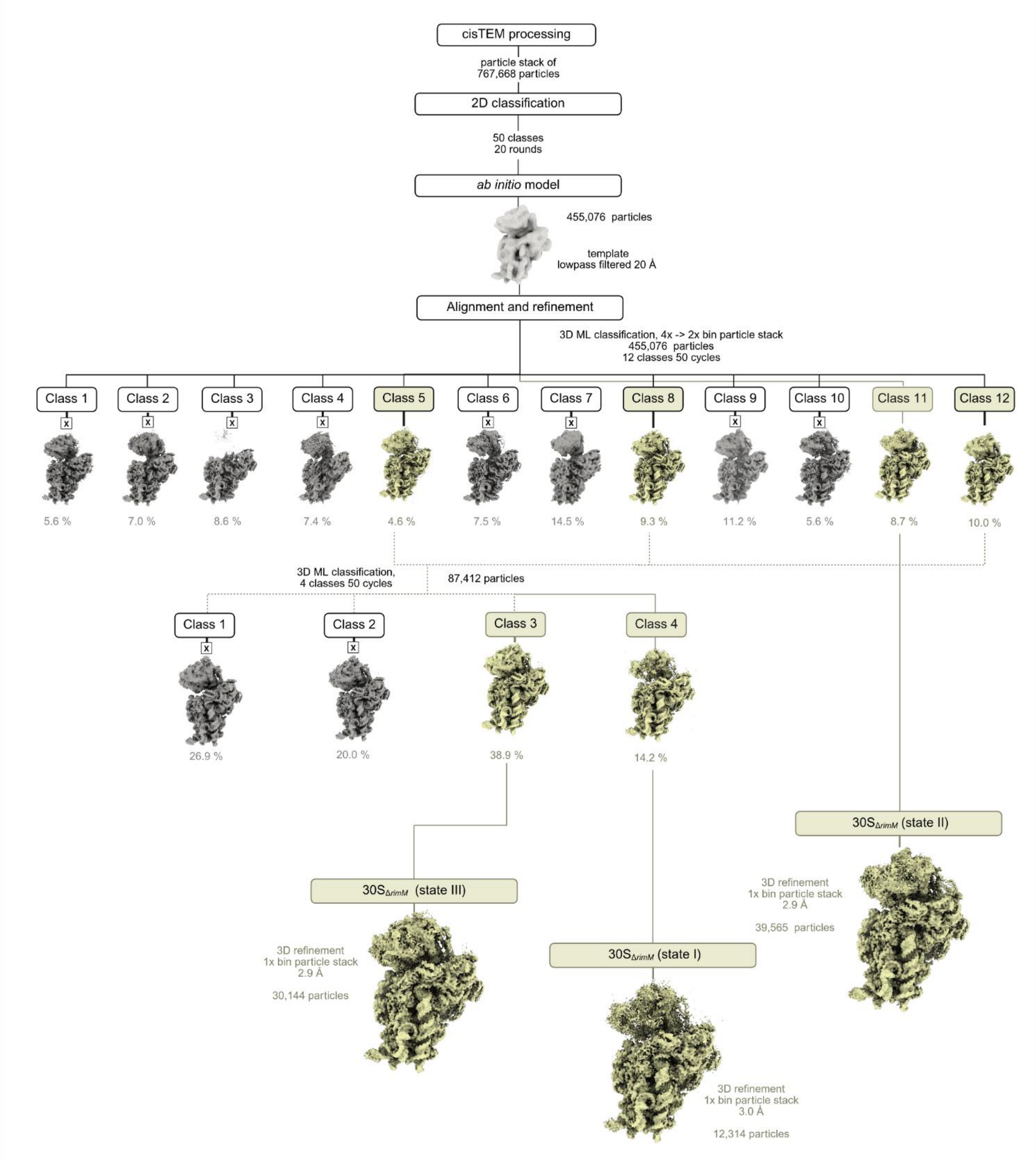

Supplementary Figure 5. Cryo-EM data processing and classification scheme for 30S$_{\Delta rimM}$ structures. Low-resolution and junk classes are shown in grey, while classes advancing to 3D classification are shown in yellow. Final maps used for structural modelling are displayed at the bottom, along with the number of particles and corresponding resolutions (FSC = 0.143).

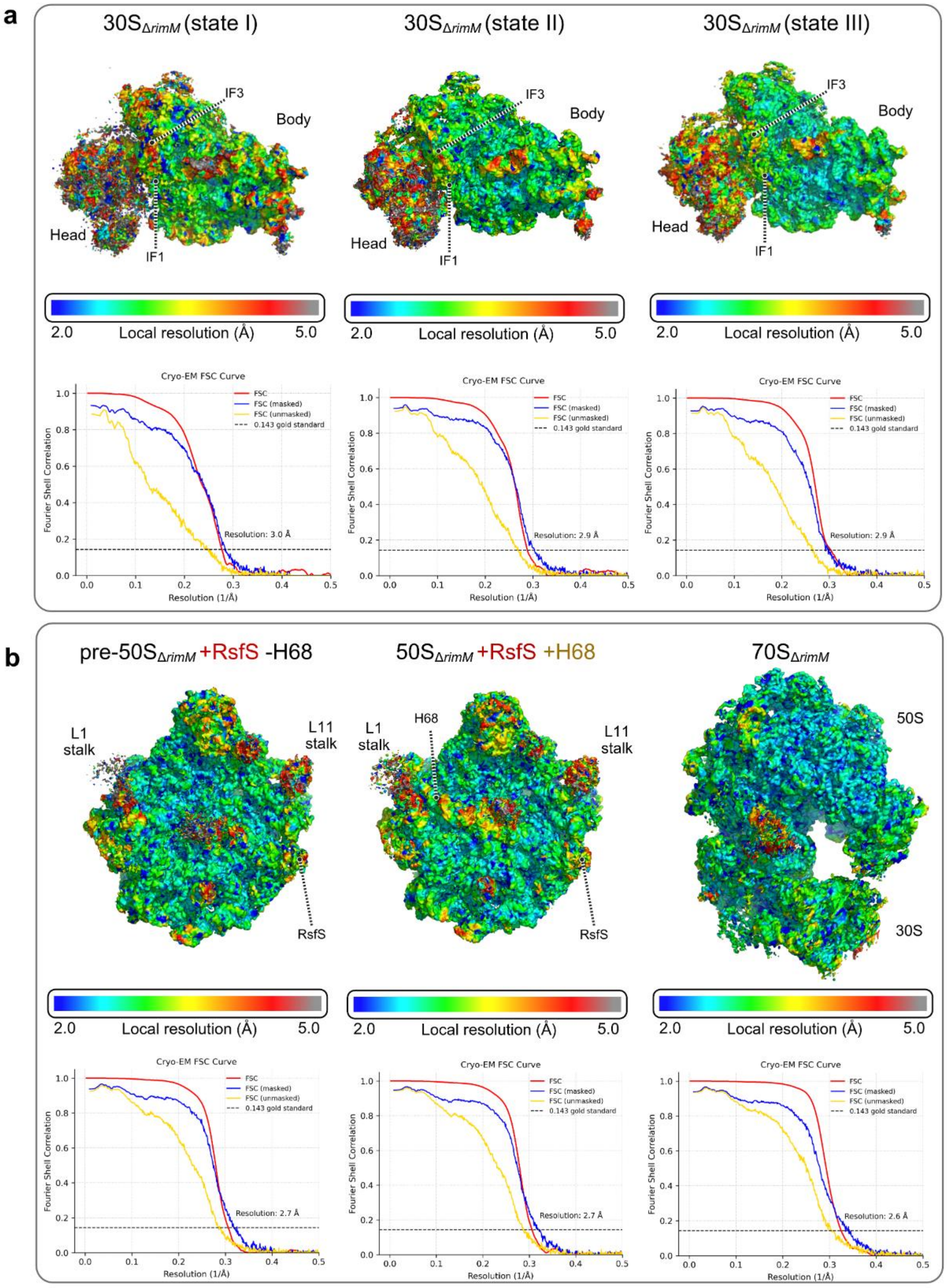

Supplementary Figure 6. Global and local resolution for Δ*rimM* 30S structures. (**a**) Local resolutions in respective $30S_{\Delta rimM}$ cryo-EM maps (top row). Fourier shell correlation (FSC) between even- and odd-particle half maps (red) reveals that map resolutions range from 2.9 to 3.0 Å (at FSC = 0.143, dotted line); FSC between final models and final maps (cyan) masked and unmasked FSCs are also shown (bottom row). (**b**) Local resolutions in respective $50S_{\Delta rimM}$ and $70S_{\Delta rimM}$ cryo-EM maps (top row). Fourier shell correlation (FSC) between even- and odd-particle half maps (red) reveals that map resolutions range from 2.9 to 3.0 Å (at FSC = 0.143, dotted line); FSC between final models and final maps (cyan) masked and unmasked FSCs are also shown (bottom row).

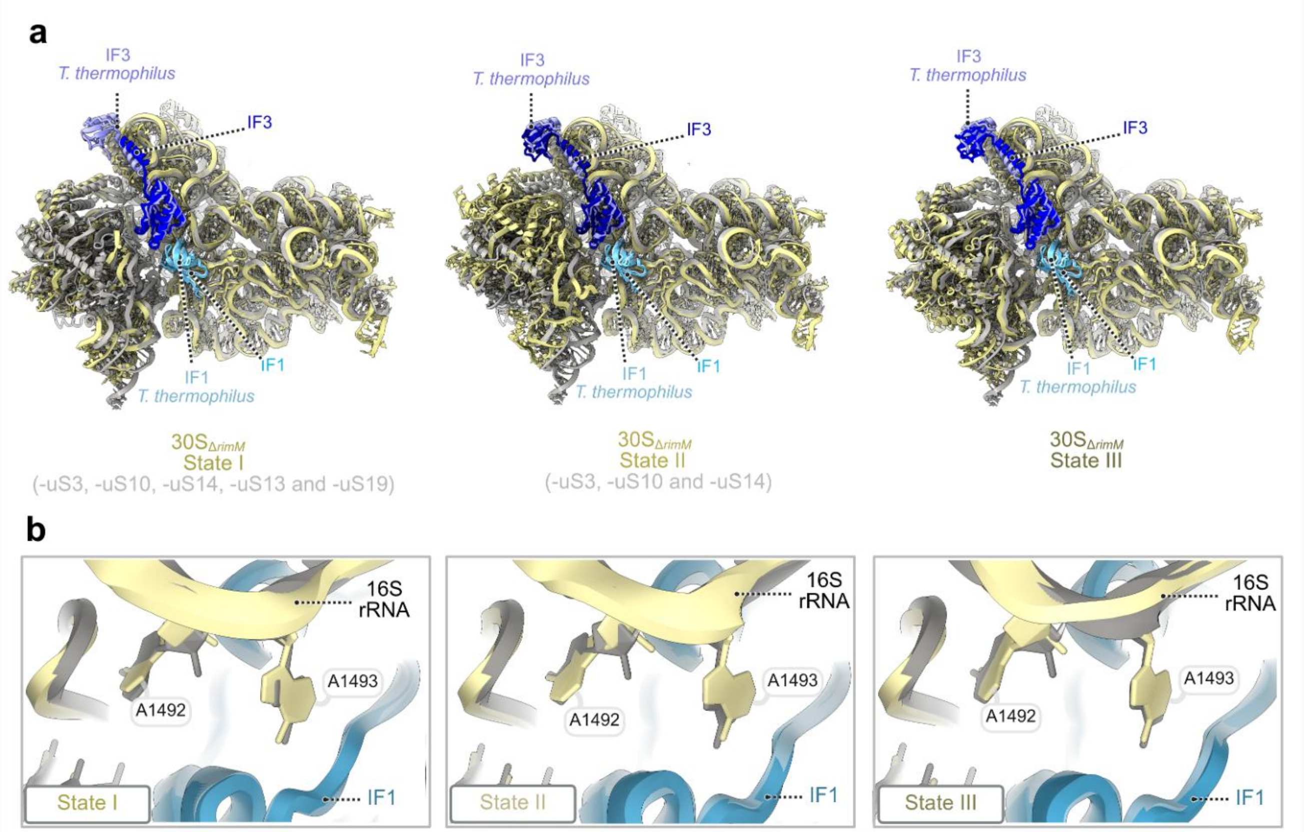

Supplementary Figure 7. Structural comparison of $30S_{\Delta RimM}$ states to the *T. thermophilus* 30S initiation complex. (a) Superposition of $30S_{\Delta rimM}$ states (I-III) with the 30S initiation complex (PDB: 5LMN) shows that IF1 and IF3 hold their canonical positions on the 30S subunit. **(b)** Close-up view of the decoding site illustrates that the arrangement of rRNA helices and decoding nucleotides A1492 and A1493 in Δ*rimM* states matches that of the initiation complex (PDB: 5LMN). Structural models of $30S_{\Delta rimM}$ subunit (state I -III) are shown in yellow, while initiation complex of *T. thermophilus* in grey (PDB: 5LMN). The initiation factors IF1 (light blue for state I, II and III and cyan for *T. thermophilus*) and IF3 (dark blue for state I, II and III and light purple for *T. thermophilus*) are also highlighted. Structural alignments were performed based on the 16S rRNA localized in the 30S body for each pairwise comparison.

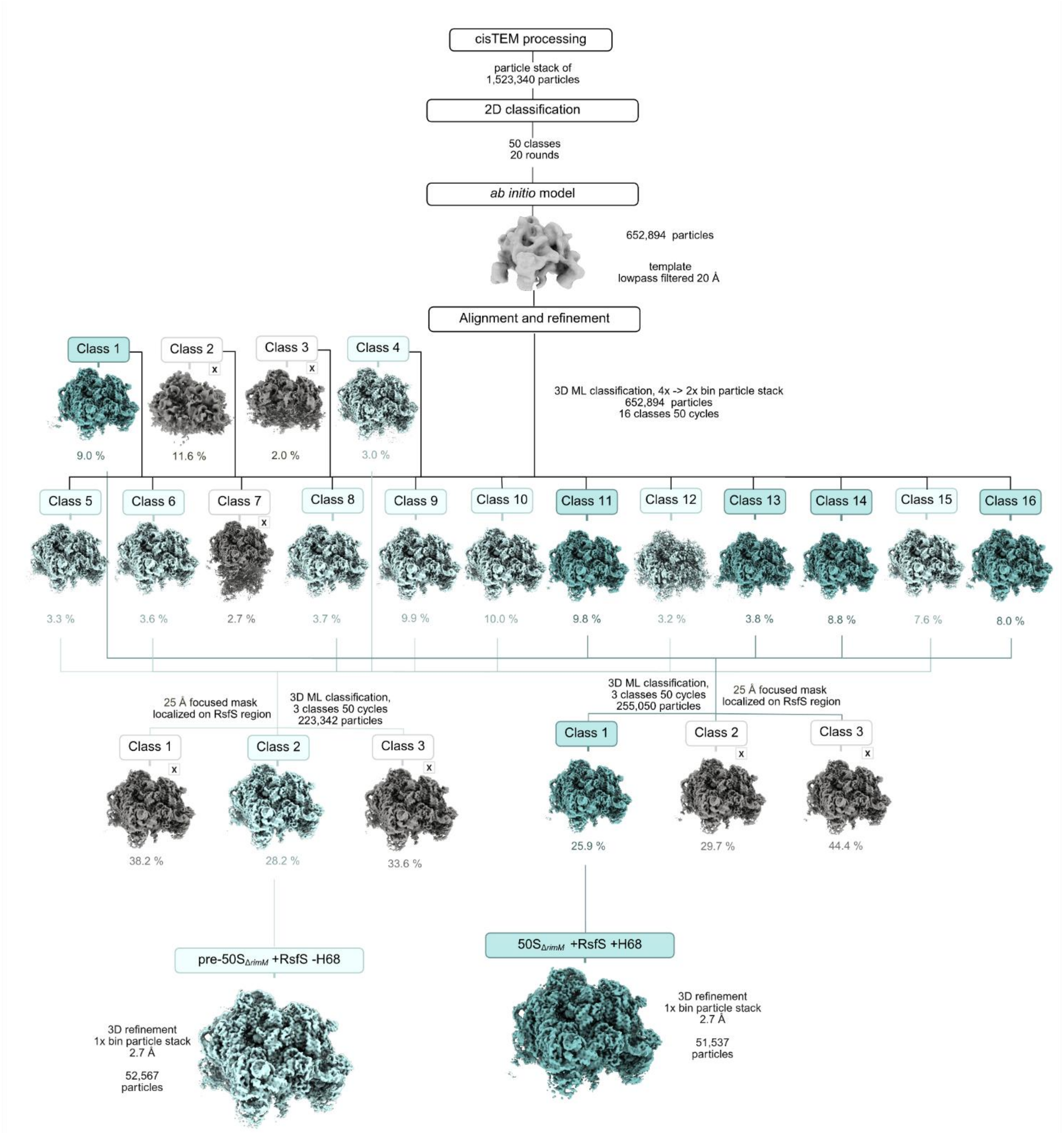

Supplementary Figure 8. Cryo-EM data processing and classification scheme for 50S$_{\Delta rimM}$ structures. Low-resolution and junk classes are shown in grey, while classes advancing to 3D classification are shown in light and dark cyan. Final maps used for structural modelling are displayed at the bottom, along with the number of particles and corresponding resolutions (FSC = 0.143).

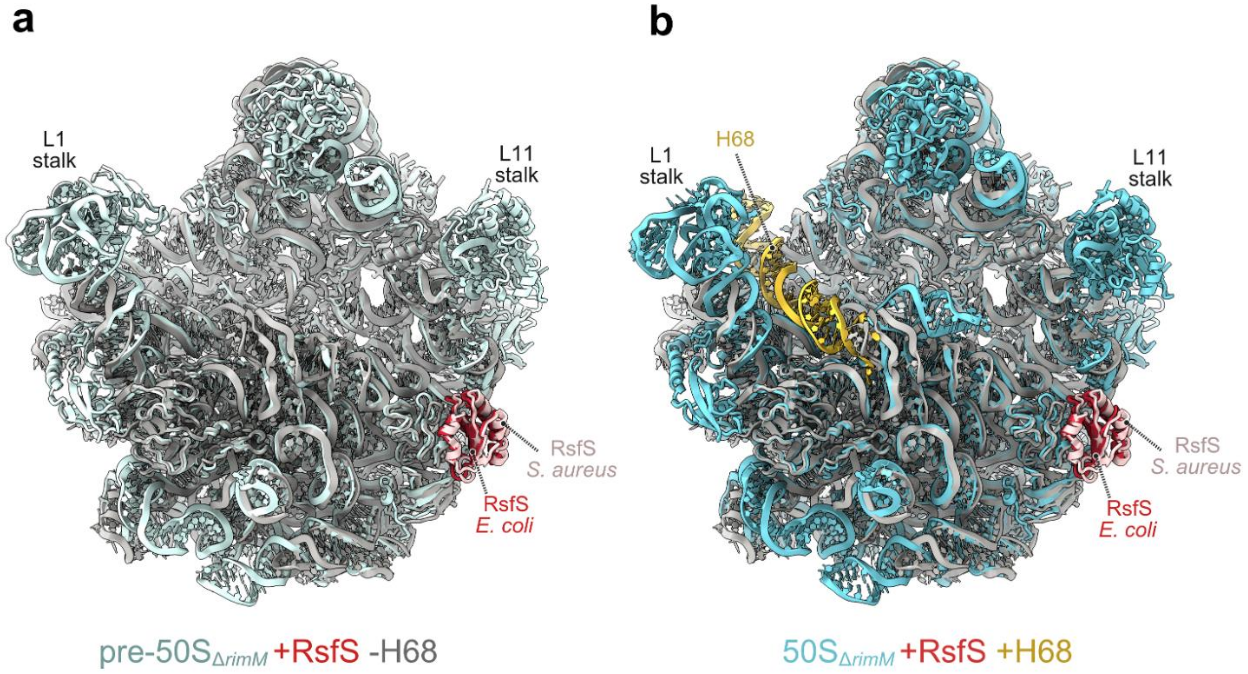

Supplementary Figure 9. Structural comparison of 50S$_{\Delta RimM}$ states to the *S. aureus* 50S-RsfS complex. (a) Superposition of pre-50S$_{\Delta rimM}$ state with the *S. aureus* 50S–RsfS complex (PDB: 6SJ6) shows consistent positioning of RsfS in the structures. **(b)** Superposition of 50S$_{\Delta rimM}$ state with the *S. aureus* 50S–RsfS complex (PDB: 6SJ6) shows consistent positioning of RsfS in the structures. The pre-50S$_{\Delta rimM}$ ribosomal subunit is shown in light blue, while 50S$_{\Delta rimM}$ is in cyan, aligned to 50S–RsfS complex from *S. aureus* in grey (PDB: 6SJ6). All structures are shown in crown view from the intersubunit interface, with RsfS highlighted in red for the 50S$_{\Delta rimM}$ states and light pink for *S. aureus* RsfS. H68 in 50S$_{\Delta rimM}$ is highlighted in gold. Structural alignments were performed based on the 23S rRNA core of the 50S subunit for each pairwise comparison.

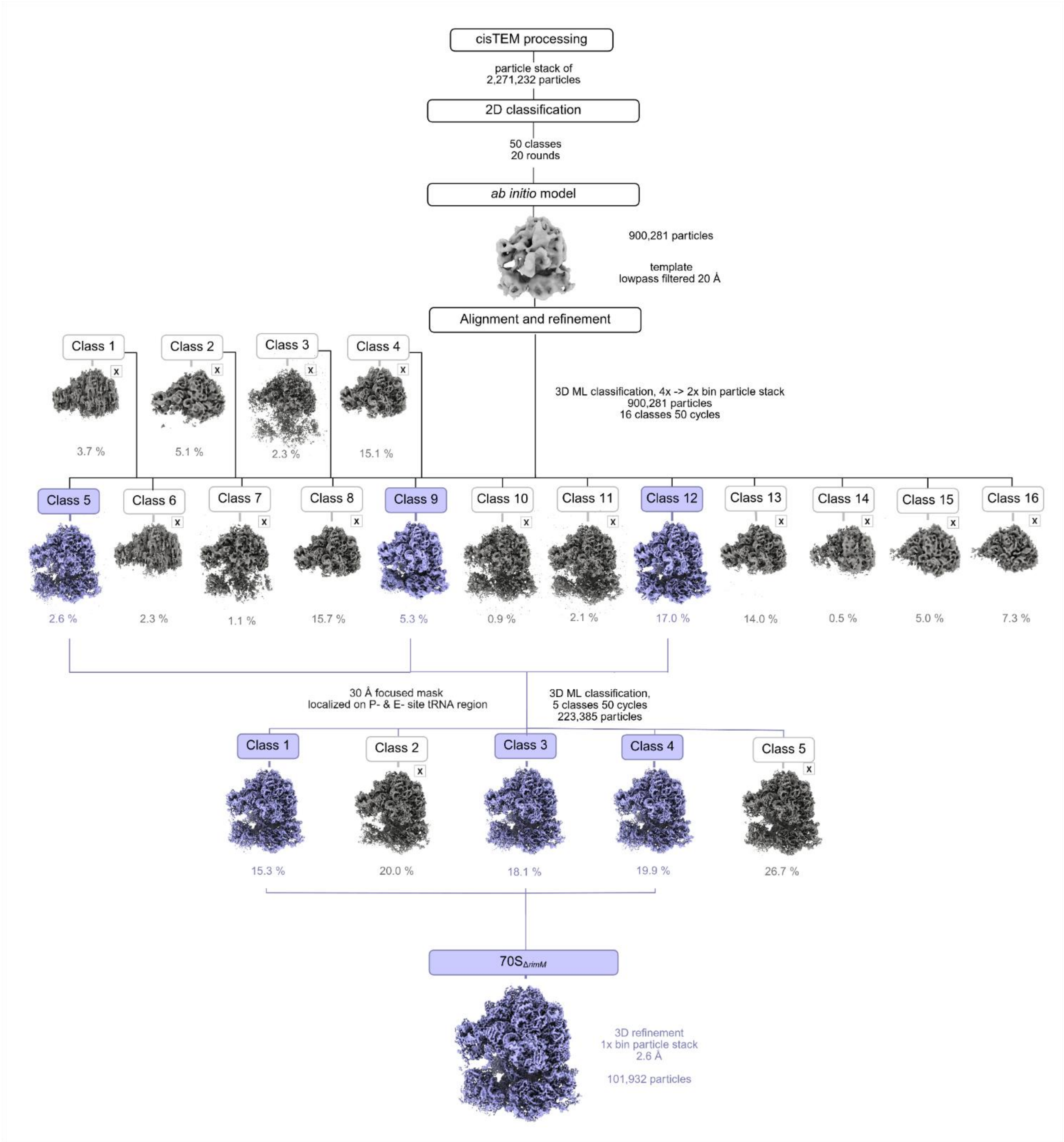

Supplementary Figure 10. Cryo-EM data processing and classification scheme for $70S_{\Delta rimM}$ structure. Low-resolution and junk classes are shown in grey, while classes advancing to 3D classification are shown in light and dark cyan. Final maps used for structural modelling are displayed at the bottom, along with the number of particles and corresponding resolutions (FSC = 0.143).

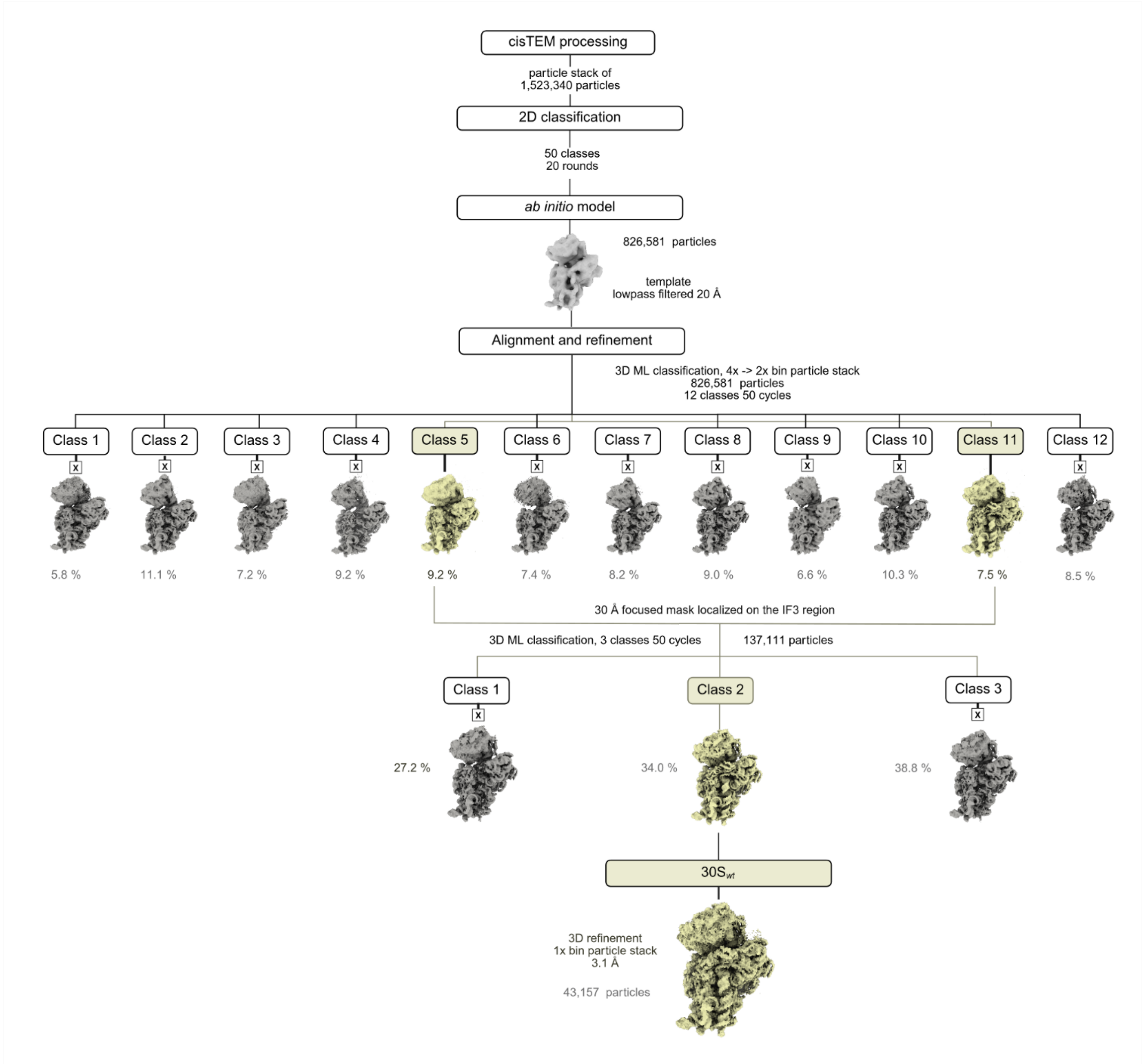

Supplementary Figure 11. Cryo-EM data processing and classification scheme for 30S$_{wt}$ structure. Low-resolution and junk classes are shown in grey, while classes advancing to 3D classification are shown in yellow. Final maps used for structural modelling are displayed at the bottom, along with the number of particles and corresponding resolutions (FSC = 0.143).

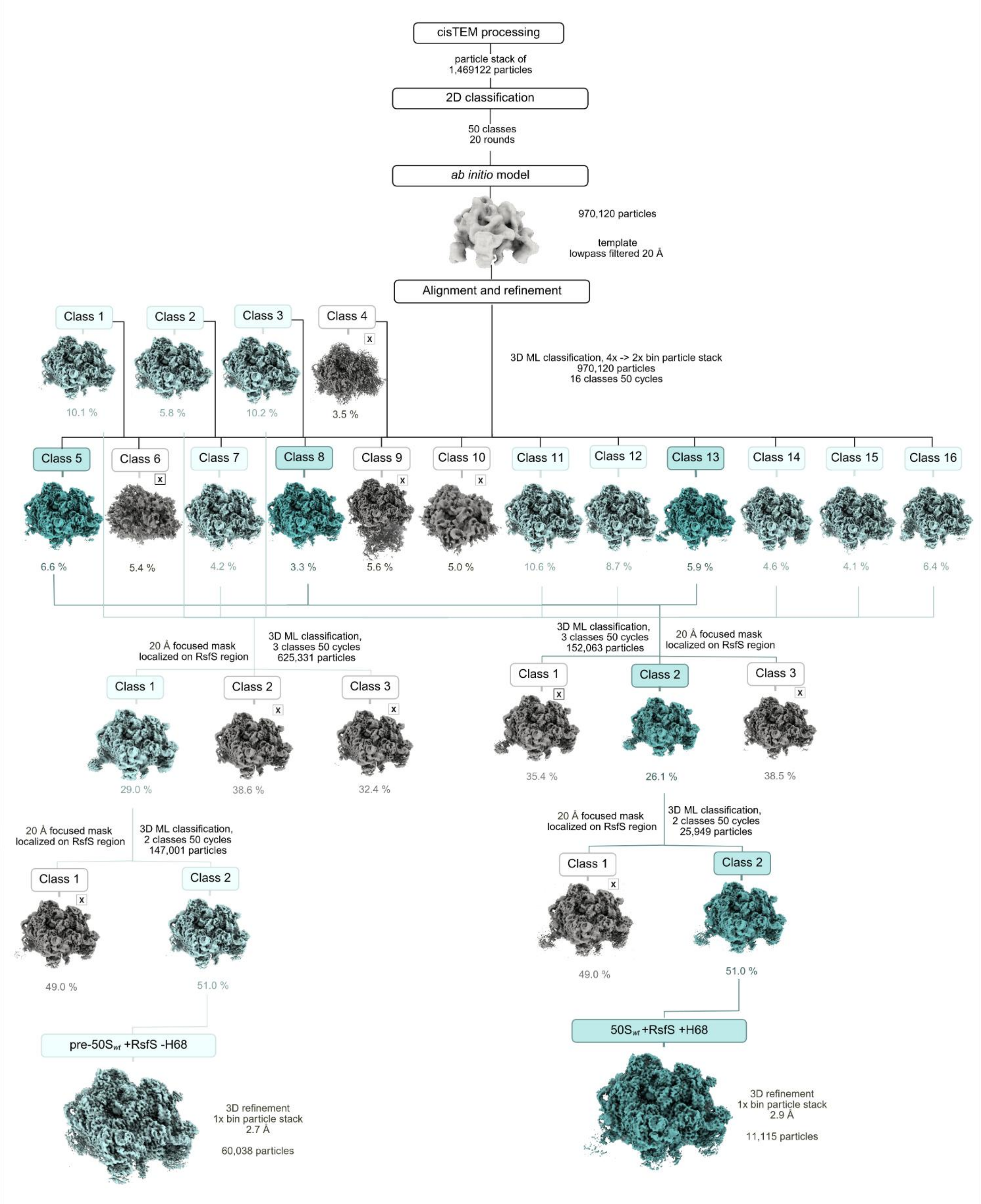

Supplementary Figure 12. Cryo-EM data processing and classification scheme for 50S$_{wt}$ structures. Low-resolution and junk classes are shown in grey, while classes advancing to 3D classification are shown in light and dark cyan. Final maps used for structural modelling are displayed at the bottom, along with the number of particles and corresponding resolutions (FSC = 0.143).

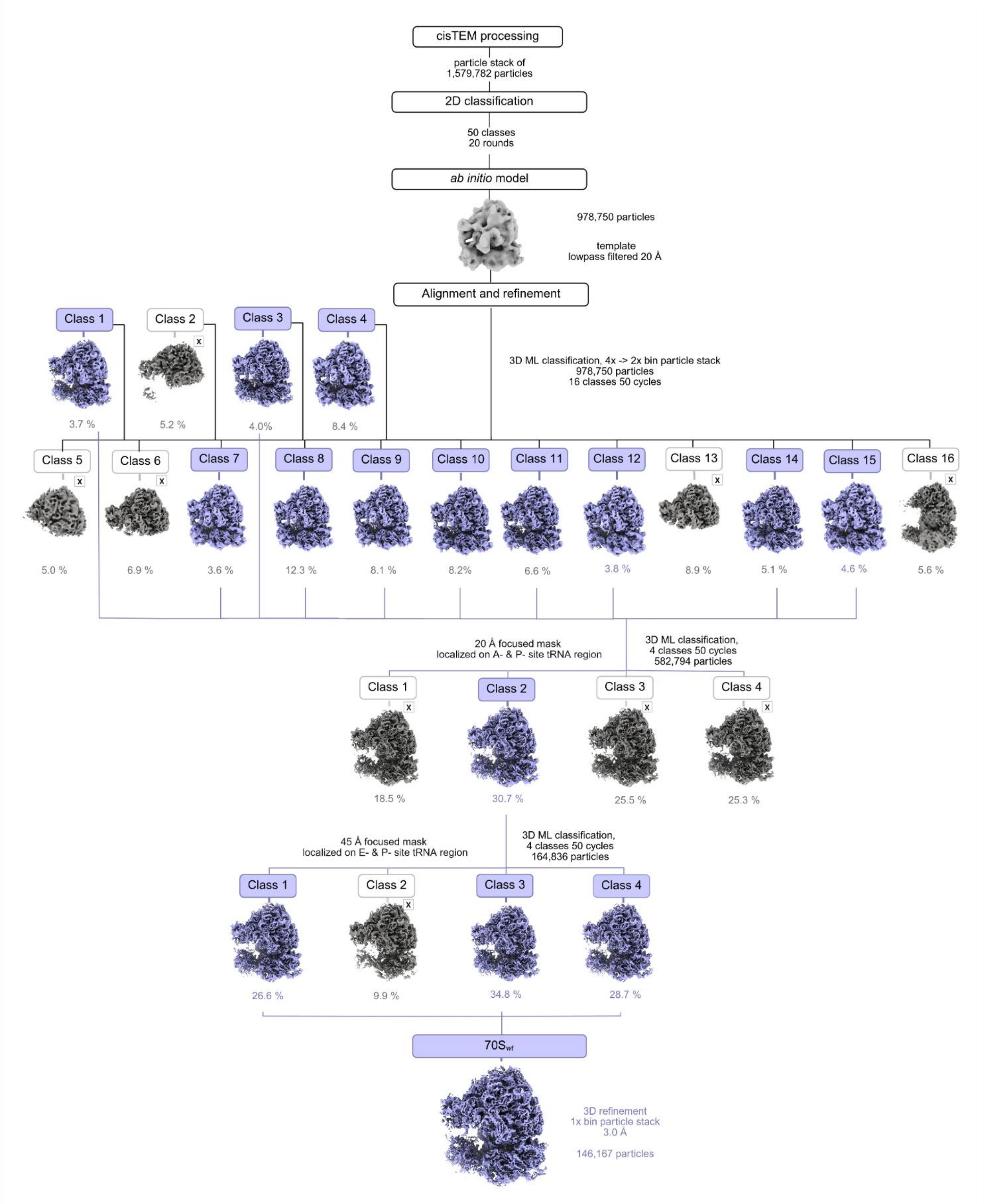

Supplementary Figure 13. Cryo-EM data processing and classification scheme for 70S$_{wt}$ structure. Low-resolution and junk classes are shown in grey, while classes advancing to 3D classification are shown in light and dark cyan. Final maps used for structural modelling are displayed at the bottom, along with the number of particles and corresponding resolutions (FSC = 0.143).

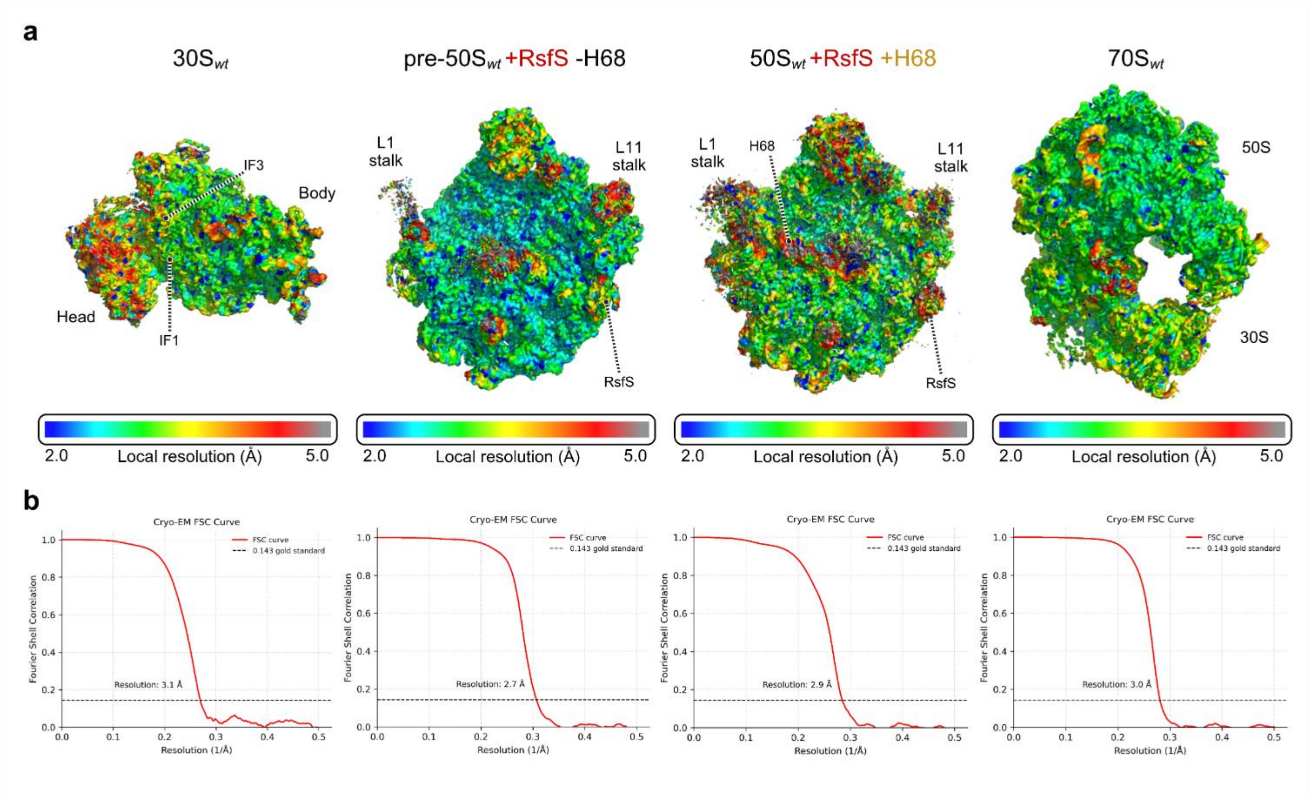

Supplementary Figure 14. Global and local resolution for wild type structures. (a) Local resolutions in respective cryo-EM maps. **(b)** Fourier shell correlation (FSC) between even- and odd-particle half maps (red) shows that map resolutions range from 2.9 to 3.0 Å (at FSC = 0.143, dotted line); FSC between final models and final maps (cyan) masked and unmasked FSCs are also shown.

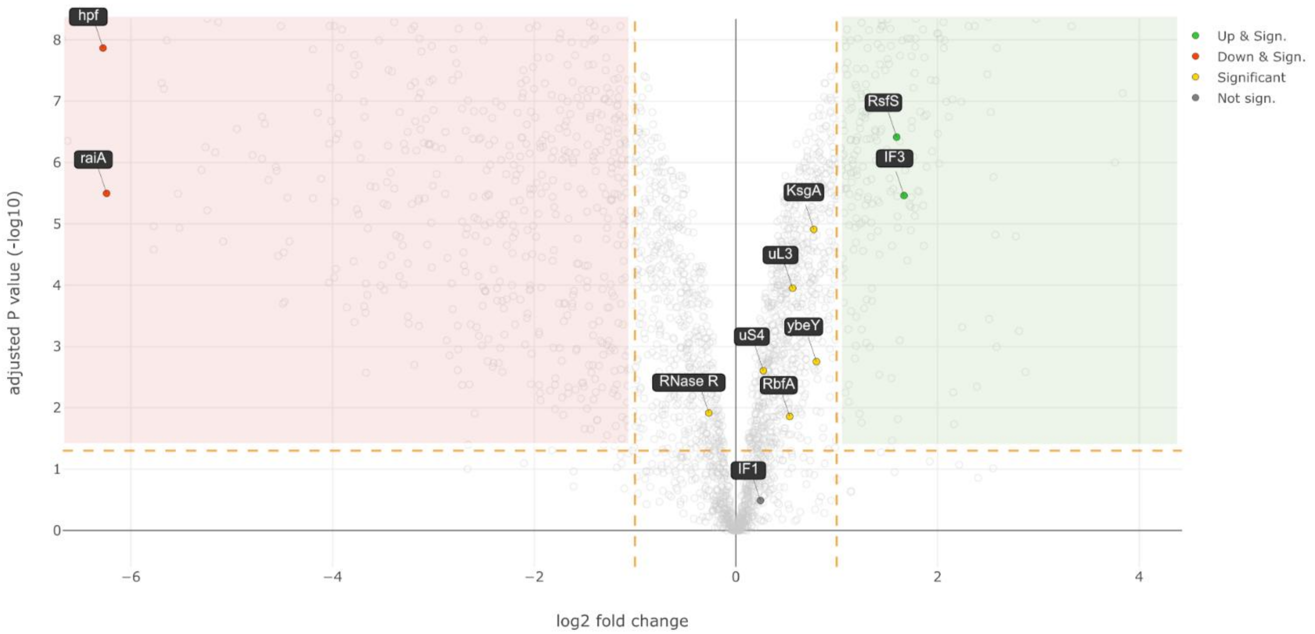

Supplementary Figure 15. Differential protein expression analysis of *wt* and Δ*rimM* strains by mass spectrometry. Volcano plot showing the $\log_2$ fold change in protein abundance (x-axis) versus the adjusted p-value ($-\log_{10}$, y-axis) for the comparison between *wt* and Δ*rimM* MRE600 strains at $OD_{600} = 0.4$. Proteins that are significantly upregulated in Δ*rimM* (adjusted p-value < 0.05, $\log_2FC > 1$) are positioned in green segment, whereas significantly downregulated proteins (adjusted p-value < 0.05, $\log_2FC < -1$) are positioned in red segment. Yellow dots represent proteins that are statistically significant but do not meet the fold-change cutoff. A filled grey dot marks a selected protein that is not statistically significant, while open grey circles represent all other detected proteins. Several ribosome-associated proteins are highlighted. RsfS and IF3 show strong upregulation (green), whereas ribosomal proteins uS4 and uL3, as well as the 30S maturation factors RbfA and KsgA, display more modest increases (yellow). In contrast, IF1 shows no significant change (grey). Notably, the ribosome hibernation factors RaiA and Hpf are strongly downregulated in the Δ*rimM* strain (red). The RNases RNase R and YbeY show only modest decreases or increases in protein abundance. The dashed lines mark the thresholds for significance.

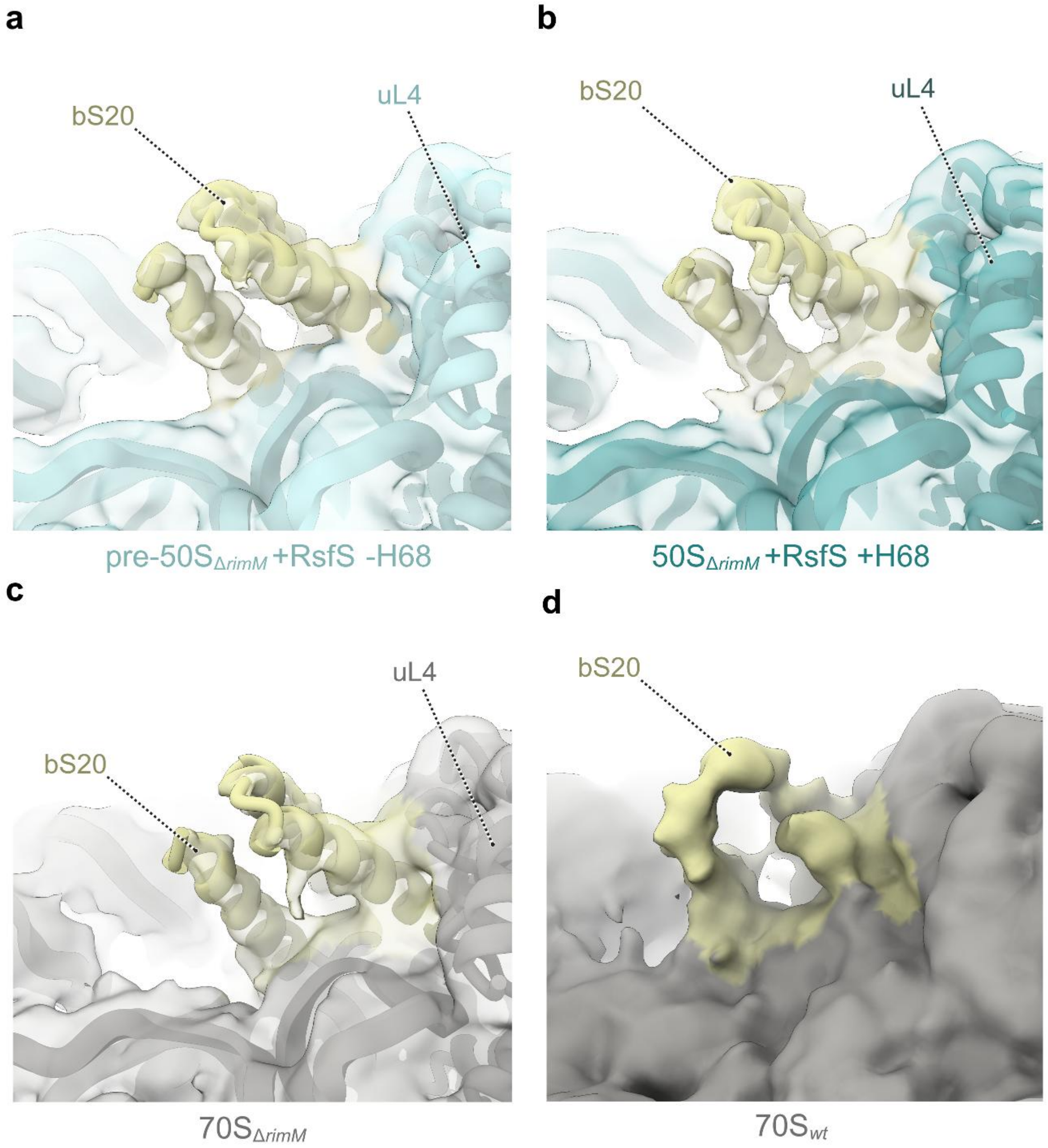

Supplementary Figure 16. Visualization of bS20 in 50S subunits and 70S ribosomes. (a) Close-up view on pre-50S$_{\Delta rimM}$ subunit (light cyan) highlighting the location of bS20 (yellow). The cryo-EM map (transparent surface) is shown at 1.5 σ. **(b)** Close-up view of the 50S$_{\Delta rimM}$ subunit (cyan) highlighting the location of bS20 (yellow). The cryo-EM map is shown at 1.5 σ. **(c)** Close-up view on the 70S$_{\Delta rimM}$ in the uL4 region of the 50S subunit (grey) highlighting the location of bS20 (yellow). The cryo-EM map is shown at 1.0 σ. **(d)** Close-up view on the 70S$_{wt}$ cryo-EM map (grey surface) highlighting the location of bS20 (yellow surface). The cryo-EM map is shown at 1.0 σ. In all cases, the cryo-EM maps were low-pass filtered applying a B-factor of 100 Å^2.
