# Supplementary material for: Mechanistic insights into E. coli recovery from growth arrest
Source: Nat Commun. 2026 Apr 11;17:5124. doi: 10.1038/s41467-026-71781-6 (PMC13246957; doi:10.1038/s41467-026-71781-6)
Supplement: Supplementary file 2 — Reporting Summary [file 41467_2026_71781_MOESM2_ESM.pdf]

nature portfolio

Corresponding author(s): Dr. Ya-Ming Hou, Dr. Gabriel Demo

Last updated by author(s): 03/06/2026

Reporting Summary

Nature Portfolio wishes to improve the reproducibility of the work that we publish. This form provides structure for consistency and transparency in reporting. For further information on Nature Portfolio policies, see our Editorial Policies and the Editorial Policy Checklist.

Statistics

For all statistical analyses, confirm that the following items are present in the figure legend, table legend, main text, or Methods section.

n/a	Confirmed	
☐	☒	The exact sample size (*n*) for each experimental group/condition, given as a discrete number and unit of measurement
☐	☒	A statement on whether measurements were taken from distinct samples or whether the same sample was measured repeatedly
☐	☒	The statistical test(s) used AND whether they are one- or two-sided<br>*Only common tests should be described solely by name; describe more complex techniques in the Methods section.*
☒	☐	A description of all covariates tested
☒	☐	A description of any assumptions or corrections, such as tests of normality and adjustment for multiple comparisons
☐	☒	A full description of the statistical parameters including central tendency (e.g. means) or other basic estimates (e.g. regression coefficient) AND variation (e.g. standard deviation) or associated estimates of uncertainty (e.g. confidence intervals)
☐	☒	For null hypothesis testing, the test statistic (e.g. *F*, *t*, *r*) with confidence intervals, effect sizes, degrees of freedom and *P* value noted<br>*Give P values as exact values whenever suitable.*
☒	☐	For Bayesian analysis, information on the choice of priors and Markov chain Monte Carlo settings
☒	☐	For hierarchical and complex designs, identification of the appropriate level for tests and full reporting of outcomes
☒	☐	Estimates of effect sizes (e.g. Cohen's *d*, Pearson's *r*), indicating how they were calculated

Our web collection on statistics for biologists contains articles on many of the points above.

Software and code

Policy information about availability of computer code

Data collection	Data collection was performed using software Serial EM (4.2.9). All software is publicly available.
Data analysis	All software used for cryo-EM data analysis has been described in Methods: cisTEM (vs 1.0 beta), Frealign v9.11, Phenix (1.21.1-5286), Chimera (vs. 1.17.3), ChimeraX (vs. 1.8), PyMol (vs 2.5.0), EMAN2 (vs 2.3.1), Bsoft (vs 1.9.1). The enzyme-based assays were analyzed using Kaleidagraph (Synergy software). All software is publicly available.

For manuscripts utilizing custom algorithms or software that are central to the research but not yet described in published literature, software must be made available to editors and reviewers. We strongly encourage code deposition in a community repository (e.g. GitHub). See the Nature Portfolio guidelines for submitting code & software for further information.

Data

Policy information about availability of data

All manuscripts must include a data availability statement. This statement should provide the following information, where applicable:

- Accession codes, unique identifiers, or web links for publicly available datasets
- A description of any restrictions on data availability
- For clinical datasets or third party data, please ensure that the statement adheres to our policy

The EM density maps generated in this study have been deposited in the EMDB under the following accession codes: EMD-55171 (30SΔrimM - State I); EMD-55173 (30SΔrimM - State II); EMD-55174 (30SΔrimM – State III); EMD-55176 (pre-50SΔrimM); EMD-55177 (50SΔrimM); EMD-55178 (70SΔrimM); EMD-55181 (30Swt); EMD-55182 (pre-50Swt); EMD-55183 (50Swt); and EMD-55185 (70Swt). The atomic coordinates generated in this study have been deposited in the PDB under the

accession codes 9SS0 (30SΔrimM - State I); 9SS1 (30SΔrimM - State II); 9SS2 (30SΔrimM – State III); 9SS4 (pre-50SΔrimM); 9SS5 (50SΔrimM); and 9SS6 (70SΔrimM). Mass spectrometry proteomics data were deposited to the ProteomeXchange Consortium via the PRIDE partner repository under dataset identifier PXD068732. For biological and enzyme-based in vitro assays source data are provided with this paper.

Research involving human participants, their data, or biological material

Policy information about studies with human participants or human data. See also policy information about sex, gender (identity/presentation), and sexual orientation and race, ethnicity and racism.

Reporting on sex and gender	N/A
Reporting on race, ethnicity, or other socially relevant groupings	N/A
Population characteristics	N/A
Recruitment	N/A
Ethics oversight	N/A

Note that full information on the approval of the study protocol must also be provided in the manuscript.

Field-specific reporting

Please select the one below that is the best fit for your research. If you are not sure, read the appropriate sections before making your selection.

☒ Life sciences ☐ Behavioural & social sciences ☐ Ecological, evolutionary & environmental sciences

For a reference copy of the document with all sections, see nature.com/documents/nr-reporting-summary-flat.pdf

Life sciences study design

All studies must disclose on these points even when the disclosure is negative.

Sample size	For biological and enzyme-based in vitro assays, a sample size of 3 and more was chosen following previously established work to evaluate the standard deviation (SD). If the SD is more than 10% of individual experiments, the sample size would be increased to 5-6, until the SD value drops down to below 10%. Cryo-EM datasets for each complex were collected such that a resolution of ~2.6 to 3.1 A could be reached. Datasets of ~12,000 to ~150, 000 particles were sufficient per each deposited state.
Data exclusions	Micrographs showing ice-contamination or aggregation were excluded from the respective cryo-EM datasets.
Replication	Replication and reproducibility were measured from the analysis of a sample size of 3 and more. All attempts of replication were successful.
Randomization	Randomization for biological and enzyme-based in vitro assays is not relevant to this study, because all samples were designed to test a hypothesis and were compared to control samples where key components of the hypothesis were maintained constant. Computational approaches to unbiased particle classification in cryo-EM (maximum likelihood classification) include randomizations. Classifications were repeated multiple times typically by varying number of classes or mask position.
Blinding	Not applicable for biological and enzyme-based in vitro assays. Blinding was not required in cryo-EM as for each sample the structural data were all analyzed using the same methods.

Reporting for specific materials, systems and methods

We require information from authors about some types of materials, experimental systems and methods used in many studies. Here, indicate whether each material, system or method listed is relevant to your study. If you are not sure if a list item applies to your research, read the appropriate section before selecting a response.

Materials & experimental systems

n/a	Involved in the study	
☒	☐	Antibodies
☒	☐	Eukaryotic cell lines
☒	☐	Palaeontology and archaeology
☒	☐	Animals and other organisms
☒	☐	Clinical data
☒	☐	Dual use research of concern
☒	☐	Plants

Methods

n/a	Involved in the study	
☒	☐	ChIP-seq
☒	☐	Flow cytometry
☒	☐	MRI-based neuroimaging

Plants

Seed stocks	N/A
Novel plant genotypes	N/A
Authentication	N/A
