# Supplementary material for: Mechanistic insights into E. coli recovery from growth arrest
Source: Nat Commun. 2026 Apr 11;17:5124. doi: 10.1038/s41467-026-71781-6 (PMC13246957; doi:10.1038/s41467-026-71781-6)
Supplement: Supplementary file 3 — Transparent Peer Review File [file 41467_2026_71781_MOESM3_ESM.pdf]

nature portfolio

Peer Review File

Mechanistic insights into E. coli recovery from growth arrest

Corresponding Author: Dr Gabriel Demo

This file contains all reviewer reports in order by version, followed by all author rebuttals in order by version.

Version 0:

Reviewer comments:

Reviewer #1

(Remarks to the Author)
In this manuscript, the authors examine the role of RimM, a protein factor previously known to contribute to the assembly of the 30S ribosomal subunit in bacteria, in the restart of growth and in restoring protein synthesis after exposure to adverse environmental conditions. They discovered that the delta RimM strain demonstrates a significantly longer growth arrest than other deletion strains lacking 30S subunit assembly factors (such as RbfA, KsgA, RlmN, RsmE, etc.). Structural analysis of the 30S subunits purified from the delta RimM strain revealed an accumulation of 30S subunits in an immature form, missing proteins of the head domain (uS13, uS10, uS19, uS3, and uS14). Interestingly, these particles are bound to IF1 and IF3. The authors propose that these two initiation factors prevent the premature association of these immature 30S subunits with 50S subunits. They also conducted structural analysis of the 50S subunits in the delta RimM strain and compared them to those in the wild-type strain. They found that, generally, the 50S subunits in the delta RimM strain are mature, except for a subset that still exhibits H68 in an immature state. A large proportion of 50S subunits in the delta RimM strain are bound to the silencing factor RsfS, and the authors suggest this may be part of the mechanism to prevent premature association of the 50S subunits with the immature 30S particles.

Overall, this is a well-conceived study on the role of RimM, an important assembly factor in bacteria. The quality of the biochemical, genetic, and structural analyses conducted by the authors is excellent, but I believe the number of novel contributions in the study does not justify publication in a general-audience journal such as Nature Communications. The research offers new insights into the molecular mechanisms of RimM's action and elaborates on aspects previously known but lacking detailed analysis or characterization. While these remain valuable contributions to the field of bacterial ribosome biogenesis, they may appeal to a more specialized audience. Below, I review some of these findings and share my perspective on their novelty and significance.

1. The initial part of the results examining the role of RimM in growth recovery is not novel. Publications from the Wikstrom group from the early 2000s (Bylund et al. (2001) J.Bact. 183:6095-6106) already describe that the delta RimM strain exhibits a seven-fold reduced growth rate and decreased translation efficiency. This new study confirms Wikstrom’s previous findings and extends them by comparing the growth rates of the delta RimM strain with those of other deletion strains lacking different 30S assembly factors.

2. The cryo-EM analysis of the 30S subunits purified from the delta RimM strain is certainly a welcome expansion of similar studies done in the early 2010s from the Gao (Guo et al. (2013) NAR 41:2609-2620) and Ortega (Leong et al. (2013) RNA 19:789-802) groups. These earlier cryo-EM studies were done before the recent dramatic technological advances of cryo-EM, and the overall resolution of the structures in those studies is limited. The present study characterizes at the molecular level the structural deficiencies of the immature 30S subunits that accumulate in the delta RimM strain. Therefore, this new study adds significant granularity to these maturation steps of the head domain assisted by RimM. In my view, the most relevant finding of this study is the finding that these assembly intermediates contain IF1 and IF3 bound to the subunit interface, preventing their association with 50S subunits.

3. The introduction section that discusses the maturation of the 50S subunit and other assembly factors should be removed because it is not relevant. This paragraph mentions the role of the ribosome silencing factor RsfS, which binds to uL14 in the 50S subunit. However, it is already known that RimM does not contribute to 50S subunit maturation. Additionally, the observed increase in RsfS-bound 50S particles in the delta RimM strain is not specific to RimM, as RsfS is known to downregulate translation during stress or stationary phase (Hauser et al., 2012). Therefore, the higher proportion of RsfS-bound 50S subunits in the delta RimM strain likely results from cells recovering from slow growth, rather than a direct effect

or link to RimM's role in maturation. Consequently, the depiction of the 50S subunit bound to RsfS in Fig. 6 as part of the recovery mechanism mediated by RimM is confusing and unsupported by the data.

4. The sequence of events involving multiple assembly factors contributing to the assembly of the 30S subunit described in Fig. 1a is highly speculative and unnecessary. The figure legend does not explain the rationale, prior knowledge, or references considered for this panel. It should be removed.

5. Suppl. Fig. 10 illustrates the classification strategy of the 30S subunit cryo-EM images obtained from the delta RimM strain. For unexplained reasons, the authors discard many classes that seem to have good resolution and may represent classes of interest. Focusing only on the refinement of the few classes highlighted in yellow could bias the interpretation of this data and, most importantly, ignore relevant biological information regarding the role of RimM contained within those classes. The authors should also consider analysing all these other classes (in grey).

6. There is an error in the x-axis label of Fig 2e; 0.14 is likely to be 0.1?

Reviewer #2

(Remarks to the Author)
In this study, Hassan et al. investigate the mechanism by which deletion of rimM, encoding a ribosome assembly factor, caused delayed recovery of E. coli cells from growth arrest. Using a combination of genetic, biochemical, and structural approaches, the authors first show that loss of rimM leads to a pronounced extension of the lag phase and a reduction in growth rate, more severe than deletions of other ribosome maturation factors. They also observe increased levels of rRNA in the ΔrimM strain. Biochemical assays using an in vitro reconstituted translation system demonstrate that ribosomes isolated from ΔrimM cells exhibit impaired translocation during translation elongation. Cryo-EM analysis further reveals that both mature and immature 30S subunits accumulate in the ΔrimM strain. The immature subunits exhibit an incompletely formed head domain, consistent with the reduced translocation efficiency observed in vitro. Although previous structural studies of ΔrimM ribosomes existed, they were of limited resolution. This work therefore provides higher-resolution structural models of 30S maturation intermediates and yields mechanistic insights into the stepwise assembly of r-proteins promoted by RimM. The authors further show that IF1 and IF3 bind more frequently to 30S subunits isolated from the ΔrimM strain, with notable binding even to immature 30S particles. These observations raise the possibility that initiation factors may participate in or influence 30S subunit maturation.

Overall, the manuscript is clearly written and easy to follow. The experiments are carefully controlled, and the combination of complementary methodologies strengthens the conclusions.

Major Comment

The authors describe RimM regulates ribosome recovery from growth arrest (e.g., lines 179 and 181). I am not fully convinced that the terms “regulate/regulator” are appropriate here. Efficient ribosome maturation by RimM appears to be a housekeeping function, not a regulatory one. Using “regulate” may suggest a regulatory role analogous to that by ribosome hibernation factors, which modulate ribosome activity in response to environmental cues, for example.

Minor Comments

Line 129:
The notation “E. coli ΔrimM (MG1655)” is ambiguous. It can sound that MG1655 is the name of the ΔrimM strain, although this point becomes clear later in the text.

If there is a specific reason why ribosome purification was performed using MRE600 rather than an MG1655 derivative, it may be helpful to briefly explain the rationale.

Reviewer #3

(Remarks to the Author)
I am not an expert in structural biology, therefore I will not comment on the Cryo-EM part of the manuscript. I focus my review on the structure supporting experiments.

In the manuscript entitled ‘Mechanistic insights into E. coli recovery from growth arrest’ by Hassan et al. authors investigate the role of RimM ribosome biogenesis factor using Cryo-EM and in vivo and in vitro experiments.
The authors conclude that cells lacking ribosomes processed by RimM protein take a longer time, compared to the wild-type cells, to enter the logarithmic growth phase. They present a prolonged lag phase. Experiments presented in the manuscript are performed correctly.

However, I have some questions about the conclusions.
1. Authors discuss recovery from growth arrest but they do not spend any time investigating what actually happened in the stationary phase to the ribosomes from delta-rimM cells. This is crucial and this is where they should look into in this manuscript.

2. Cells in a delta-rimM strain take longer to restart growth. What happens to the ribosomes in the stationary phase? Is rRNA degraded, truncated? Do ribosomes have problems hibernating to sustain their function? Authors should check the stability of rRNA in the stationary phase, for example using capillary gel electrophoresis (like for example, Bioanalyser). Is rRNA more prone for degradation in the stationary phase? Based on the structure, can you predict rRNA degradation (if confirmed)?

3. Do ribosomes in delta-rimM form 100S hibernation complexes (compare to the wt)? Based on the structure, can you predict if the binding of the hibernation factors will be disturbed? Are hibernation factors downregulated in MS? (I am aware the data is not from the stationary phase).

4. Sucrose gradient profiles from the stationary phase, and OD 0.1 and 1 are missing. Stationary phase data and OD 0.1 would be crucial to determine what happened to the ribosomes in the stationary phase.

5. Mass spec data is not available; authors should provide a dataset in the submission set (for example Excel dataset).

6. Authors use OD600 to normalize cell density for comparative experiments (for example, RNA, protein content per cell). Have the authors checked the relation of OD and colony-forming units? Cells with different size scatter light differently.

Version 1:

Reviewer comments:

Reviewer #1

(Remarks to the Author)
I appreciate the revisions these authors made in response to my feedback and comments. In the revised version and the point-by-point response letter, it is now clear how this new study significantly expands earlier work from the Wikstrom lab on the slow-growth phenotype of the delta RimM strain. Based on their responses, I agree that this paper represents a significant expansion of this particular aspect, including a systematic comparison of this phenotype with strains lacking other 30S biogenesis factors and/or ribosomal proteins. None of this has been reported before. Similarly, cryo-EM analysis of the 30S subunits purified from the delta RimM strain significantly expands our structural understanding of these ribosomal particles compared with lower-resolution studies around 2013 from the Guo and Ortega groups. I also welcome the changes the authors have made regarding their interpretation of the 50S subunits purified from the delta RimM strain and their association with RsfS. I think the text describing these findings is now well adjusted and consistent with the existing literature on RsfS.

Overall, all my concerns are addressed, and I am supportive of the publication of this revised version of the manuscript as is.

Reviewer #2

(Remarks to the Author)
The authors have addressed all my comments.

Reviewer #3

(Remarks to the Author)
The reply to my comments by authors of the the manuscript entitled ‘Mechanistic insights into E. coli recovery from growth arrest’ by Hassan et al. is satisfactory.
This is a complex, comprehensive work which should be published.

Minor comment:
line 312 RsfS controls the 50S subunit association and 70S assembly
Association with? it should be written down.

Reviewer #4

(Remarks to the Author)
LC-MS/MS Technical Review: Mechanistic insights into E. coli recovery from growth arrest

Hassan et. al use LC-MS/MS to probe the differential expression between wild type E. coli and E. coli ΔrimM strain, which exhibits a prolonged growth arrest. Their work complements other broader experimentation. The researchers use a modern

Bruker Tims TOF HT mass spectrometer with a nanoLC and long gradient and a DIA scanning method. The hardware is up to date, and the DIA parameters are standard and easily reproduced on such an instrument. Three biological replicates are used, and the sample prep follows established protocols. The data is also mined using standard search methods and follows a logical progression of protocols and statistics to arrive at the result of protein expression differences. As such, the overall LC-MS/MS technical methods are current and accurate.

Minor issues with the technical experimentation are as follows:

(1) The supplemental information in PRIDE contains 12 raw mass spectrometry files and the sample information supports that. However, the experimental section is based on 2 strains in triplicate, so the question is why are the additional raw files provided?

(2) Authors use iRT + trypsin database presumably for retention correction/normalization. However, it is not written in the sample prep if these iRT peptides are spiked in or not. As such, clarification is warranted.

(3) In the statistical write up section of the LC-MS/MS methods the authors should include: what is the method used for multiple testing correction (eg. Benjamini-Hochberg, other), what is the cut off for significant protein FDR and fold change and which category of proteins were filtered in or out and annotated in the volcano plot. To this effect, a list of all proteins found should be provided in PRIDE.

(4) The coloring scheme of the Volcano plot is very light and hard to read. A more contrasting color palette would make it easier for readers.

Open Access This Peer Review File is licensed under a Creative Commons Attribution 4.0 International License, which permits use, sharing, adaptation, distribution and reproduction in any medium or format, as long as you give appropriate credit to the original author(s) and the source, provide a link to the Creative Commons license, and indicate if changes were made.
In cases where reviewers are anonymous, credit should be given to 'Anonymous Referee' and the source.
The images or other third party material in this Peer Review File are included in the article's Creative Commons license, unless indicated otherwise in a credit line to the material. If material is not included in the article's Creative Commons license and your intended use is not permitted by statutory regulation or exceeds the permitted use, you will need to obtain permission directly from the copyright holder.
To view a copy of this license, visit https://creativecommons.org/licenses/by/4.0/

Point-by-point response to the reviewer comments

Reviewer #1 (Remarks to the Author):
Overall, this is a well-conceived study on the role of RimM, an important assembly factor in bacteria. The quality of the biochemical, genetic, and structural analyses conducted by the authors is excellent, but I believe the number of novel contributions in the study does not justify publication in a general-audience journal such as Nature Communications. The research offers new insights into the molecular mechanisms of RimM's action and elaborates on aspects previously known but lacking detailed analysis or characterization. While these remain valuable contributions to the field of bacterial ribosome biogenesis, they may appeal to a more specialized audience. Below, I review some of these findings and share my perspective on their novelty and significance.

1. The initial part of the results examining the role of RimM in growth recovery is not novel. Publications from the Wikstrom group from the early 2000s (Bylund et al. (2001) J.Bact. 183:6095-6106) already describe that the delta RimM strain exhibits a seven-fold reduced growth rate and decreased translation efficiency. This new study confirms Wikstrom's previous findings and extends them by comparing the growth rates of the delta RimM strain with those of other deletion strains lacking different 30S assembly factors.
We appreciate the reviewer's comments regarding the growth recovery phenotype of bacterial strain lacking the 30S biogenesis factor RimM. While we acknowledge that a slow-growth phenotype of Δ*rimM* strain has been reported previously, we believe the novelty of our study lies in the systematic comparison of this phenotype with strains lacking other 30S biogenesis factors and/or ribosomal proteins. This direct comparison reveals that loss of RimM produces the most severe recovery defect among the factors examined, underscoring the critical importance of proper maturation and folding of the 3′ head domain of the 30S subunit for efficient translation. Notably, deletion of the primary head-domain ribosomal protein uS9 or of RbfA—often considered a central player in 30S biogenesis—does not result in a comparable phenotype. In addition, by analyzing ribosomes isolated from lag, early exponential, and late exponential growth phases, we provide mechanistic insight into why defects in the 30S head domain must be resolved to restore efficient translocation of tRNAs and mRNA during recovery. We believe that these combined functional insights offer a comprehensive perspective on the role of RimM in 30S biogenesis and are well suited for a broad readership, such as that of *Nature Communications*.

2. The cryo-EM analysis of the 30S subunits purified from the delta RimM strain is certainly a welcome expansion of similar studies done in the early 2010s from the Gao (Guo et al. (2013) NAR 41:2609-2620) and Ortega (Leong et al. (2013) RNA 19:789-802) groups. These earlier cryo-EM studies were done before the recent dramatic technological advances of cryo-EM, and the overall resolution of the structures in those studies is limited. The present study characterizes at the molecular level the structural deficiencies of the immature 30S subunits that accumulate in the delta RimM strain.

Therefore, this new study adds significant granularity to these maturation steps of the head domain assisted by RimM. In my view, the most relevant finding of this study is the finding that these assembly intermediates contain IF1 and IF3 bound to the subunit interface, preventing their association with 50S subunits.

We thank the reviewer for this assessment and for placing our work in the context of the pioneering studies by the Gao and Ortega groups. We fully agree that these earlier investigations provided important initial insights into 30S assembly intermediates in the absence of RimM; however, they were necessarily limited by both resolution and scope, and focused exclusively on characterizing 30S maturation defects. To our knowledge, no previous study has attempted to comprehensively address how cells ultimately assemble functional 70S ribosomes and restore translation under conditions of perturbed 30S biogenesis.

The central advance of our study lies precisely in this broader perspective. By resolving, at near-atomic resolution, a complete and internally consistent set of ribosomal particles—30S, 50S, and 70S—isolated from the Δ*rimM* background, we move to directly interrogate how ribosome assembly, quality control, and translational recovery are coordinated. Importantly, parallel analysis of ribosomal particles isolated from wild-type cells provides a critical reference framework that allows us to distinguish defective and fully functional states, and to directly assess how productive 70S ribosomes are assembled despite impaired 30S maturation.

Within this framework, our cryo-EM analysis resolves discrete maturation states of the 30S subunit that capture progressive folding of the 3′ major domain of the 16S rRNA and stepwise incorporation of late-binding head-domain ribosomal proteins. While some structural features are consistent with earlier reports, the improved resolution enables localization of rRNA disorder, protein occupancy, and factor binding, allowing these intermediates to be placed into a functional context that was previously inaccessible.

Importantly, and as highlighted by the reviewer, we consider the most significant finding of this work to be the association of IF1 and IF3 with immature 30S particles. Our structures show that these initiation factors remain bound at the subunit interface across multiple maturation states, thereby preventing premature association with the 50S subunit. This provides a direct structural mechanism explaining how cells avoid the formation of defective 70S ribosomes while still permitting recovery of translational capacity once properly assembled subunits become available.

Crucially, this anti-association checkpoint was not resolved, despite similar purification strategies, and likely reflects both the improved resolving power of current cryo-EM methodologies and our deliberate focus on ribosomal particles isolated during early exponential recovery. By integrating high-resolution structural analysis with functional assays, quantitative proteomics, and growth-phase–resolved ribosome profiling, our study offers a comprehensive view of how bacteria preserve translational fidelity and restore protein synthesis when 30S biogenesis is perturbed. We believe that this integrated conceptual and methodological framework provides a broadly applicable strategy for investigating translation recovery and ribosome quality control under stress conditions and will be of interest to a broad readership.

3. The introduction section that discusses the maturation of the 50S subunit and other assembly factors should be removed because it is not relevant. This paragraph mentions the role of the ribosome silencing factor RsfS, which binds to uL14 in the 50S subunit. However, it is already known that RimM does not contribute to 50S subunit maturation. Additionally, the observed increase in RsfS-bound 50S particles in the delta RimM strain is not specific to RimM, as RsfS is known to downregulate translation during stress or stationary phase (Hauser et al., 2012). Therefore, the higher proportion of RsfS-bound 50S subunits in the delta RimM strain likely results from cells recovering from slow growth, rather than a direct effect or link to RimM's role in maturation. Consequently, the depiction of the 50S subunit bound to RsfS in Fig. 6 as part of the recovery mechanism mediated by RimM is confusing and unsupported by the data.

We thank the reviewer for this important comment and agree that RimM is not directly involved in 50S subunit maturation. In response, we have removed the introductory text describing 50S biogenesis and late-stage 50S assembly factors, as it was not essential for the central focus of the study.

We have revised the remaining text to clarify that our interest in the 50S subunit does not concern its maturation, but rather whether perturbation of 30S biogenesis elicits a coordinated cellular response that limits premature subunit joining. Accordingly, the discussion of RsfS has been reformulated to emphasize its established role as a stress-responsive anti-association factor, rather than implying a RimM-specific function in 50S assembly.

Consistent with prior work (including Hauser et al., 2012), we now explicitly state that the increased occupancy of RsfS observed in the Δ*rimM* strain reflects a general adaptive response to slow growth, rather than a direct mechanistic link between RimM and RsfS. In this context, deletion of RimM induces prolonged recovery and impaired 30S maturation, which in turn triggers upregulation of anti-association mechanisms—including RsfS—to prevent premature formation of defective 70S ribosomes.

We have also revised Figure 6 and its legend to avoid suggesting that RsfS acts as part of a RimM-specific recovery pathway. Instead, the figure now illustrates how RsfS functions alongside IF1 and IF3 as part of a broader, stress-induced safeguarding program that restrains 70S assembly when 30S biogenesis is compromised.

Together, these revisions clarify that our findings do not attribute a novel role of RimM in 50S maturation, but rather demonstrate how impaired 30S assembly engages conserved anti-association pathways to ensure that only properly matured ribosomal subunits enter translation.

4. The sequence of events involving multiple assembly factors contributing to the assembly of the 30S subunit described in Fig. 1a is highly speculative and unnecessary. The figure legend does not explain the rationale, prior knowledge, or references considered for this panel. It should be removed.

In response to the reviewer's comment, Figure 1a has been removed to avoid speculative or potentially misleading interpretation of the presented data.

5. Suppl. Fig. 10 illustrates the classification strategy of the 30S subunit cryo-EM images obtained from the delta RimM strain. For unexplained reasons, the authors discard many classes that seem to have good resolution and may represent classes of interest. Focusing only on the refinement of the few classes highlighted in yellow could bias the interpretation of this data and, most importantly, ignore relevant biological information regarding the role of RimM contained within those classes. The authors should also consider analysing all these other classes (in grey).
We understand the concern raised by the reviewer and would like to clarify how these classes were handled. We carefully and systematically analyzed all additional particle classes shown in grey to avoid bias or omission in our interpretation of 30S maturation in the absence of RimM. Many of these classes were merged into smaller stacks and subjected to multiple rounds of focused 3D classification, using masks targeting either the 30S head domain or the mRNA tunnel, in an effort to identify any additional structurally or biologically relevant states. However, these classes consistently recapitulated features of 30S maturation intermediates that have already been described in earlier studies, including those reported by Guo et al., NAR, (2013) and Leong et al., RNA, (2013), as noted in the Results section. We therefore focused our analysis on additional classes that are particularly relevant to the control of 70S assembly, as these states not only reflect sequential ribosomal protein incorporation but also reveal the involvement of initiation factors acting as anti-association elements. Notably, such states were not resolved in previous studies, despite similar purification strategies. By collecting 30S samples at OD_{600} = 0.4, our dataset captures high-resolution structural states that were previously omitted and that provide new insight into a mechanism that safeguards 70S assembly.

6. There is an error in the x-axis label of Fig 2e; 0.14 is likely to be 0.1?
We thank the reviewer for noting this typo error; it has been corrected in panel e of Figure 2.

Reviewer #2 (Remarks to the Author):
In this study, Hassan et al. investigate the mechanism by which deletion of rimM, encoding a ribosome assembly factor, caused delayed recovery of E. coli cells from growth arrest. Using a combination of genetic, biochemical, and structural approaches, the authors first show that loss of rimM leads to a pronounced extension of the lag phase and a reduction in growth rate, more severe than deletions of other ribosome maturation factors. They also observe increased levels of rRNA in the ΔrimM strain. Biochemical assays using an in vitro reconstituted translation system demonstrate that ribosomes isolated from ΔrimM cells exhibit impaired translocation during translation elongation. Cryo-EM analysis further reveals that both mature and immature 30S subunits accumulate in the ΔrimM strain. The immature subunits exhibit an incompletely formed head domain, consistent with the reduced translocation efficiency observed in vitro. Although previous structural studies of ΔrimM ribosomes existed, they were of limited resolution. This work therefore provides higher-resolution structural models of 30S maturation intermediates and yields mechanistic insights into

the stepwise assembly of r-proteins promoted by RimM. The authors further show that IF1 and IF3 bind more frequently to 30S subunits isolated from the ΔrimM strain, with notable binding even to immature 30S particles. These observations raise the possibility that initiation factors may participate in or influence 30S subunit maturation.

Overall, the manuscript is clearly written and easy to follow. The experiments are carefully controlled, and the combination of complementary methodologies strengthens the conclusions.

Major Comment

The authors describe RimM regulates ribosome recovery from growth arrest (e.g., lines 179 and 181). I am not fully convinced that the terms “regulate/regulator” are appropriate here. Efficient ribosome maturation by RimM appears to be a housekeeping function, not a regulatory one. Using “regulate” may suggest a regulatory role analogous to that by ribosome hibernation factors, which modulate ribosome activity in response to environmental cues, for example.

We thank the reviewer for this important note. We agree that the terms “regulate” and “regulator” may imply a conditional or stress-responsive role that is not appropriate for RimM. RimM functions as a constitutive 30S biogenesis factor required for efficient ribosome maturation, rather than as a regulatory factor that modulates ribosome activity in response to environmental cues, such as ribosome hibernation factors.

Accordingly, we have revised the manuscript to remove or replace “regulate/regulator” where it referred to RimM. These instances have been rephrased to describe RimM as being “required for,” or “supporting” efficient ribosome maturation and recovery from growth arrest. This revised wording more accurately reflects RimM’s housekeeping role in 30S assembly and avoids conflating its function with stress-responsive regulatory mechanisms.

Minor Comments

Line 129:

The notation “E. coli ΔrimM (MG1655)” is ambiguous. It can sound that MG1655 is the name of the ΔrimM strain, although this point becomes clear later in the text.

We thank the reviewer for pointing this out. We have revised the wording to clearly state that the Δ*rimM* was constructed in the *E. coli* MG1655 background, thereby avoiding any confusion that MG1655 might refer to the mutant strain name itself.

If there is a specific reason why ribosome purification was performed using MRE600 rather than an MG1655 derivative, it may be helpful to briefly explain the rationale.

We have added a brief explanation in the Results section describing the structural analysis of 30S intermediates, clarifying that ribosome purification was performed using the *E. coli* MRE600 background because of its reduced ribonuclease activity, which may facilitate preservation of 30S assembly intermediates and is also relevant for subsequent purification and functional validation of 70S ribosomes in kinetic assays.

Reviewer #3 (Remarks to the Author):

I am not an expert in structural biology, therefore I will not comment on the Cryo-EM part of the manuscript. I focus my review on the structure supporting experiments.

In the manuscript entitled 'Mechanistic insights into E. coli recovery from growth arrest' by Hassan et al. authors investigate the role of RimM ribosome biogenesis factor using Cryo-EM and in vivo and in vitro experiments.
The authors conclude that cells lacking ribosomes processed by RimM protein take a longer time, compared to the wild-type cells, to enter the logarithmic growth phase. They present a prolonged lag phase. Experiments presented in the manuscript are performed correctly.

However, I have some questions about the conclusions.
1. Authors discuss recovery from growth arrest but they do not spend any time investigating what actually happened in the stationary phase to the ribosomes from delta-rimM cells. This is crucial and this is where they should look into in this manuscript.
We thank the reviewer for raising this important point and fully agree that understanding ribosome behavior during stationary phase in the Δ*rimM* background is biologically highly relevant. The primary goal of the present study, however, is to define how cells restore functional translation and productive 70S ribosome assembly upon exit from growth arrest when 30S biogenesis is perturbed by loss of RimM.
Accordingly, our study focuses on the recovery phase, during which ribosomes transition from inactive or immature states to translationally competent 70S complexes. This window enabled us to identify a structural and functional checkpoint characterized by delayed 30S head-domain maturation and sustained binding of anti-association factors (IF1, IF3, and RsfS), which together prevent premature or defective 70S assembly. We therefore deliberately concentrated on this phase to comprehensively demonstrate, using complementary structural, biochemical, and kinetic approaches, how the translation machinery is restored under conditions of impaired 30S biogenesis. However, as pointed out by the reviewer, the ribosome remodeling during stationary phase represents a distinct and biologically important question. We have already begun investigating this growth phase in the Δ*rimM* strain; however, our intention is to address it as a separate study in which we can comprehensively examine how ribosome hibernation is altered or reprogrammed under conditions of impaired 30S biogenesis. Such an analysis will require an integrated structural and biochemical approach to define the mechanisms that govern translational silencing and preservation during stationary phase.
In the present manuscript, which focuses on translation reactivation during the recovery phase, we therefore limit our analysis of stationary phase to polysome profiling across multiple growth stages (Supplementary Fig. 4). These data reveal that the hibernation scheme is markedly altered in Δ*rimM* cells, highlighting the need for further detailed investigation into the factors and mechanisms that control this

reprogramming, which is currently ongoing in our lab.

2. Cells in a delta-rimM strain take longer to restart growth. What happens to the ribosomes in the stationary phase? Is rRNA degraded, truncated? Do ribosomes have problems hibernating to sustain their function? Authors should check the stability of rRNA in the stationary phase, for example using capillary gel electrophoresis (like for example, Bioanalyser). Is rRNA more prone for degradation in the stationary phase? Based on the structure, can you predict rRNA degradation (if confirmed)?
We fully agree with the reviewer that assessing rRNA stability and degradation during stationary phase in the Δ*rimM* strain is an important direction. In the current study, we did not perform direct measurements of rRNA integrity in stationary phase as our primary focus was on the recovery phase and the structural mechanisms enabling reactivation of translation.
That said, our stationary phase polysome profiles (Supplementary Fig. 4) reveal reduced formation of hibernating 100S complexes and an altered balance of free 30S and 50S subunits in Δ*rimM* cells, suggesting that ribosome preservation during stationary phase may indeed be compromised. In the Discussion, we cautiously postulate that this phenotype could be linked to a pool of incompletely matured 30S subunits—particularly those with defects in the 3′ end of the 16S rRNA—which may be more susceptible to ribonuclease-mediated turnover.
This hypothesis is further supported by our proteomic data (Supplementary Fig. 15) showing downregulation of canonical hibernation factors (HPF, RaiA) during early exponential growth, potentially predisposing ribosomes entering stationary phase to reduced protection. However, we fully agree that direct analysis of rRNA stability and degradation in stationary phase is required to validate this model. Such experiments, including biochemical assays of rRNA integrity, are a major focus of our ongoing work and are intentionally separated from the recovery-focused narrative of the present manuscript.

3. Do ribosomes in delta-rimM form 100S hibernation complexes (compare to the wt)? Based on the structure, can you predict if the binding of the hibernation factors will be disturbed? Are hibernation factors downregulated in MS? (I am aware the data is not from the stationary phase).
We thank the reviewer for raising this important question. Polysome profiling demonstrates that Δ*rimM* cells are capable of forming 100S ribosome hibernation complexes; however, their abundance is reduced in stationary phase compared to wild-type cells, accompanied by an altered distribution of free 30S and 50S subunits (Supplementary Fig. 4). These observations are now presented and discussed in the revised manuscript.
Consistent with these findings, our mass spectrometry analysis performed during early exponential growth reveals downregulation of several canonical ribosome hibernation factors in the Δ*rimM* background. Although these measurements were not conducted in stationary phase, they suggest that ribosomes entering stationary phase in Δ*rimM* cells may already be insufficiently protected, potentially compromising the efficiency or

stability of hibernation. For clarity, we have updated the Supplementary Figure 15 to highlight hibernation and ribosome quality control associated factors, including Hpf, RaiA, RNase R, and YebY.
Based on our structural data, we do not predict direct disruption of hibernation factor binding sites on mature ribosomes. Instead, as discussed in the manuscript, we propose that incomplete 30S maturation combined with altered factor availability may indirectly affect ribosome hibernation, in line with previous observations (Prossliner *et al.*, NAR, 2021). Importantly, we note that the effects of rimM deletion are not limited to hibernation alone, but are also likely to influence ribosome collisions and associated quality control pathways.
We emphasize that these interpretations are made cautiously. A comprehensive understanding of how disrupted 30S biogenesis impacts ribosome hibernation and quality control will require extensive structural analyses as well as complementary biochemical and rRNA degradation studies. As noted, such investigations are currently underway in our laboratories to generate a full comprehensive story.

4. Sucrose gradient profiles from the stationary phase, and OD 0.1 and 1 are missing. Stationary phase data and OD 0.1 would be crucial to determine what happened to the ribosomes in the stationary phase.
We agree with the reviewer that growth-phase resolved ribosome profiles are important. We have therefore included polysome profiling data from Δ*rimM* cells harvested at OD_{600} = 0.1 (termed lag/recovery phase), OD_{600} = 1.0 (termed late exponential phase), and stationary phase (Supplementary Fig. 4). These data demonstrate that ribosome distributions at OD_{600} = 0.1 and 1.0 closely resemble those observed at OD_{600} = 0.4, validating that the structural analyses performed at OD_{600} = 0.4 are representative. In contrast, stationary phase profiles show a distinct redistribution of ribosomal species, particularly affecting 100S formation and the balance of free subunits. We now explicitly present and discuss these data prior to the structural description of 30S intermediates, which we identify as an important avenue for future investigation.

5. Mass spec data is not available; authors should provide a dataset in the submission set (for example Excel dataset).
We have now included the complete processed mass spectrometry datasets as Excel table and deposited it as a raw source MS file for access directly linked to this manuscript.

6. Authors use OD600 to normalize cell density for comparative experiments (for example, RNA, protein content per cell). Have the authors checked the relation of OD and colony-forming units? Cells with different size scatter light differently.
We thank the reviewer for raising this important point regarding the use of OD_{600} as a proxy for cell number. First, to address the concern that differences in cell size between *wt* and Δ*rimM* strains might affect the relationship between OD_{600} and cell number, we directly compared colony-forming units (CFUs) at matched OD_{600} values. As shown in Supplementary Fig. 1I, cultures of *wt* and Δ*rimM* strains were collected at OD_{600} ≈ 0.4

during growth at 37°C, diluted, and plated on LB agar. At the same dilution (10^{-4}), both strains yielded comparable numbers of colonies, indicating similar CFU per unit OD_{600}. This demonstrates that, under these conditions, OD_{600} reflects comparable numbers of viable cells in *wt* and Δ*rimM* liquid cultures. Differences in colony size and plate incubation time reflect differences in growth rate on solid medium and do not affect the CFU-based estimation of cell number in liquid culture. Second, with regard to the use of OD_{600} for normalization, we would like to clarify that OD_{600} was not used to normalize cellular RNA or protein content across samples collected at different optical densities. Instead, OD_{600} was used solely to monitor growth and to harvest cells at defined growth phases. All comparative analyses of RNA and protein content were performed on samples collected at comparable growth stages, thereby avoiding normalization across different OD conditions. Additionally, a clarification sentence was added in the viability assay method section.

Point-by-point response to the reviewer comments

Reviewer #1 (Remarks to the Author):

I appreciate the revisions these authors made in response to my feedback and comments. In the revised version and the point-by-point response letter, it is now clear how this new study significantly expands earlier work from the Wikstrom lab on the slow-growth phenotype of the delta RimM strain. Based on their responses, I agree that this paper represents a significant expansion of this particular aspect, including a systematic comparison of this phenotype with strains lacking other 30S biogenesis factors and/or ribosomal proteins. None of this has been reported before. Similarly, cryo-EM analysis of the 30S subunits purified from the delta RimM strain significantly expands our structural understanding of these ribosomal particles compared with lower-resolution studies around 2013 from the Guo and Ortega groups. I also welcome the changes the authors have made regarding their interpretation of the 50S subunits purified from the delta RimM strain and their association with RsfS. I think the text describing these findings is now well adjusted and consistent with the existing literature on RsfS.

Overall, all my concerns are addressed, and I am supportive of the publication of this revised version of the manuscript as is.

We thank the reviewer for the constructive remarks throughout the review process and appreciate the opportunity to address all of the concerns raised.

Reviewer #2 (Remarks to the Author):

The authors have addressed all my comments.

We thank the reviewer for the constructive remarks throughout the review process and appreciate the opportunity to address all of the concerns raised.

Reviewer #3 (Remarks to the Author):

The reply to my comments by authors of the the manuscript entitled ‘Mechanistic insights into E. coli recovery from growth arrest’ by Hassan et al. is satisfactory. This is a complex, comprehensive work which should be published.

Minor comment:
line 312 RsfS controls the 50S subunit association and 70S assembly Association with? it should be written down.

We thank the reviewer for the constructive remarks throughout the review process. The subheading has been revised to: “RsfS controls the association of the 50S subunit with the 30S subunit and the 70S assembly.”

Reviewer #4 (Remarks to the Author):

LC-MS/MS Technical Review: Mechanistic insights into E. coli recovery from growth arrest

Hassan et. al use LC-MS/MS to probe the differential expression between wild type E. coli and E. coli ΔrimM strain, which exhibits a prolonged growth arrest. Their work complements other broader experimentation. The researchers use a modern Bruker Tims TOF HT mass spectrometer with a nanoLC and long gradient and a DIA scanning method. The hardware is up to date, and the DIA parameters are standard and easily reproduced on such an instrument. Three biological replicates are used, and the sample prep follows established protocols. The data is also mined using standard search methods and follows a logical progression of protocols and statistics to arrive at the result of protein expression differences. As such, the overall LC-MS/MS technical methods are current and accurate.

Minor issues with the technical experimentation are as follows:

(1) The supplemental information in PRIDE contains 12 raw mass spectrometry files and the sample information supports that. However, the experimental section is based on 2 strains in triplicate, so the question is why are the additional raw files provided?

We thank the reviewer for raising this point and apologize for the lack of clarity in the original description. In this study, two *E. coli* strains - *Escherichia coli* K-12 MG1655 (MG1655) and *Escherichia coli* MRE600 (MRE600)- were analyzed in biological triplicates. For each strain, two variants were examined (*wt* and Δ*rimM*), resulting in a total of 12 LC–MS/MS measurements (2 strains × 2 variants × 3 biological replicates). Accordingly, 12 raw files were generated and deposited in PRIDE. We have now

revised the Methods section to explicitly describe this experimental design. In the manuscript, we present only the volcano plot derived from the MRE600 dataset (*wt* vs. Δ*rimM*). This is because the cryo-EM experiments of ribosomal particles were performed using the MRE600 strain, and we therefore focused on this dataset to ensure a direct correlation between the structural and proteomic analyses. The MG1655 dataset was nevertheless deposited in PRIDE to support transparency and open science. Although it shows comparable trends and is not directly linked to the structural work presented here, we consider it valuable to make these raw data publicly available for the scientific community.

(2) Authors use iRT + trypsin database presumably for retention correction/normalization. However, it is not written in the sample prep if these iRT peptides are spiked in or not. As such, clarification is warranted.

We thank the reviewer for pointing out this omission. We agree that the use of iRT peptides was not described with sufficient clarity in the original sample preparation section. The Methods section has now been revised to explicitly state that iRT peptides were spiked into each sample prior to LC–MS/MS analysis.

(3) In the statistical write up section of the LC-MS/MS methods the authors should include: what is the method used for multiple testing correction (eg. Benjamini-Hochberg, other), what is the cut off for significant protein FDR and fold change and which category of proteins were filtered in or out and annotated in the volcano plot. To this effect, a list of all proteins found should be provided in PRIDE.

The Methods section has been revised to explicitly describe the statistical procedures used for the LC–MS/MS analysis. Specifically, we now state the method used for multiple testing correction, the false discovery rate (FDR) cutoff applied for significance, and the fold-change threshold used to define differential protein abundance. Regarding the volcano plot, we apologize for the previously unclear labeling. The grey open circles do not represent filtered-out proteins; rather, they correspond to all other proteins detected by the MS analysis that are not specifically

highlighted in the figure. These proteins were not individually annotated because they are not directly relevant to the main focus of the manuscript. We have now clarified this in the legend of Supplementary Figure 15, where it is explicitly stated that grey circles represent all other detected proteins, while only proteins relevant to this study are highlighted in colored dots and labeled. Finally, in line with the reviewer's request and to ensure full data transparency, the complete list of all detected proteins has been deposited in the PRIDE repository and is also provided as a source data file accompanying the manuscript.

(4) The coloring scheme of the Volcano plot is very light and hard to read. A more contrasting color palette would make it easier for readers.

We thank the reviewer for this helpful suggestion. To improve the clarity and readability of the volcano plot, we have revised the color scheme in Supplementary Figure 15. Specifically, we increased the contrast of the color palette by using darker colors for the data points and open circles, and we relabelled the key proteins using labels with a black background. These changes enhance the visibility of the highlighted proteins and make the figure easier to interpret, particularly for the proteins that are most relevant to the conclusions of this manuscript.
